# Discovery of frameshifting in Alphavirus 6K resolves a 20-year enigma

**DOI:** 10.1186/1743-422X-5-108

**Published:** 2008-09-26

**Authors:** Andrew E Firth, Betty YW Chung, Marina N Fleeton, John F Atkins

**Affiliations:** 1 BioSciences Institute, University College Cork, Cork, Ireland; 2 Department of Microbiology, Moyne Institute for Preventive Medicine, Trinity College, Dublin 2, Ireland; 3 Department of Human Genetics, University of Utah, Salt Lake City, UT 84112-5330, USA

## Abstract

**Background:**

The genus *Alphavirus *includes several potentially lethal human viruses. Additionally, species such as Sindbis virus and Semliki Forest virus are important vectors for gene therapy, vaccination and cancer research, and important models for virion assembly and structural analyses. The genome encodes nine known proteins, including the small '6K' protein. 6K appears to be involved in envelope protein processing, membrane permeabilization, virion assembly and virus budding. In protein gels, 6K migrates as a doublet – a result that, to date, has been attributed to differing degrees of acylation. Nonetheless, despite many years of research, its role is still relatively poorly understood.

**Results:**

We report that ribosomal -1 frameshifting, with an estimated efficiency of ~10–18%, occurs at a conserved UUUUUUA motif within the sequence encoding 6K, resulting in the synthesis of an additional protein, termed TF (TransFrame protein; ~8 kDa), in which the C-terminal amino acids are encoded by the -1 frame. The presence of TF in the Semliki Forest virion was confirmed by mass spectrometry. The expression patterns of TF and 6K were studied by pulse-chase labelling, immunoprecipitation and immunofluorescence, using both wild-type virus and a TF knockout mutant. We show that it is predominantly TF that is incorporated into the virion, not 6K as previously believed. Investigation of the 3' stimulatory signals responsible for efficient frameshifting at the UUUUUUA motif revealed a remarkable diversity of signals between different alphavirus species.

**Conclusion:**

Our results provide a surprising new explanation for the 6K doublet, demand a fundamental reinterpretation of existing data on the alphavirus 6K protein, and open the way for future progress in the further characterization of the 6K and TF proteins. The results have implications for alphavirus biology, virion structure, viroporins, ribosomal frameshifting, and bioinformatic identification of novel frameshift-expressed genes, both in viruses and in cellular organisms.

## Background

The *Alphavirus *genus (reviewed in [1,2]) includes ≥29 species, many of which infect humans and livestock. Species include Sindbis virus (SINV), Semliki Forest virus (SFV), Eastern, Western and Venezuelan equine encephalitis viruses (EEEV, WEEV, VEEV), Chikungunya virus, Ross River virus (RRV), Middelburg virus (MIDV), Seal louse virus (SESV) and Sleeping disease virus (SDV). Alphavirus symptoms include infectious arthritis, rashes, fever and potentially fatal encephalitis. Transmission is generally via insects such as mosquitoes, with birds, rodents and other mammals acting as reservoirs for many species. The distribution of certain species has been expanding in recent years [3] – a phenomenon that can only be expected to continue as changing climate allows the insect vectors to expand their ranges.

**Figure 1:**
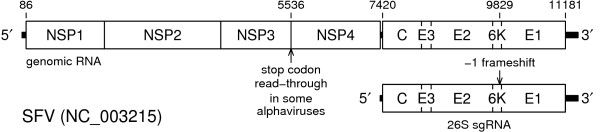
**Alphavirus genome map**. The position of the -1 ribosomal frameshift site is indicated. Nucleotide coordinates are for SFV ([GenBank:NC_003215]; 11442 nt).

The single-stranded genomic RNA is positive sense and about 11–12 kb long. It contains two long open reading frames (ORFs) separated by a short non-coding sequence (Figure 1). The 5'-proximal ORF codes for non-structural proteins and often contains an internal stop codon read-through site. The 3'-proximal ORF codes for an ~140 kDa structural polyprotein (C-E3-E2-6K-E1) that is translated from a subgenomic RNA (26S sgRNA) and cleaved autocatalytically (to generate the capsid protein C) and by cellular proteases (to yield the envelope glycoproteins E1, E2 and E3). The virion has icosahedral symmetry with *T *= 4, and comprises an inner nucleocapsid (240 copies of the capsid protein enclosing the genomic RNA) and a tight outer envelope composed of 240 copies of the envelope proteins (arranged as 80 E1-E2 heterodimer trimeric spikes) embedded in a lipid bilayer derived from the host cell membrane [1]. E3 is present in the virion of some (e.g. SFV) but not all (e.g. SINV) alphaviruses.

The 6K protein is a small, hydrophobic, cysteine-rich, acylated protein, involved in envelope protein processing, membrane permeabilization, virus budding and virus assembly – though only small amounts of 6K are actually incorporated into virions [1,4-14]. Mutations in 6K are associated with greatly decreased virion production and/or deformed multicored virions though, interestingly, 6K deletion mutants are still viable [15-23]. Although 6K was previously observed to migrate as a doublet [7,15,16,21], the potential for a ribosomal frameshift leading to two different proteins appears to have been overlooked, perhaps in part because of the one-to-one stoichiometry of the C, E3, E2 and E1 proteins in the virion. Instead the doublet was explained as a result of differing degrees of acylation [7,15].

In this paper, we describe bioinformatic analyses that allowed us to identify a frameshift site within the 6K coding sequence, and we provide experimental evidence that verifies expression of the predicted transframe protein, TF. Further characterization of the function(s) of TF is beyond the scope of this paper and will be addressed in future work. The results have implications for (*i*) alphavirus biology, (*ii*) virion structure, (*iii*) research into viroporins, (*iv*) ribosomal frameshifting, and (*v*) bioinformatic identification of novel frameshift-expressed genes, both in viruses and in cellular organisms (especially where the out-of-frame ORF is short).

## Results

### A bioinformatic search identifies a likely frameshift site

Many viruses harbour sequences that induce a portion of ribosomes to shift -1 nt and continue translating in the new reading frame [24]. The -1 frameshift site typically consists of a slippery heptanucleotide fitting the consensus motif X XXY YYZ, where X is any nucleotide, Y is A or U, and Z is not G. This is followed by a 'spacer' region of 5–9 nt, and then a highly structured region – often a pseudoknot or hairpin. We first identified the potential -1 frameshift site in the alphavirus 6K coding sequence during a systematic search of virus genome alignments for phylogenetically conserved frameshifting motifs (Firth, unpublished). The slippery site U UUU UUA (spaces separate the polyprotein or zero-frame codons) – conforming to the X XXY YYZ consensus – is conserved in 353 of the 357 alphavirus sequences in GenBank that contain the 6K coding sequence (see methods for accession numbers of all 357 sequences). This alone is highly significant since amino acid conservation in the polyprotein frame only requires conservation of three of these nucleotides. Interestingly, the same U UUU UUA motif is used at the Gag-Pol -1 frameshift site in all Human immunodeficiency virus type 1 (HIV-1) groups, besides other primate lentiviruses.

**Figure 2:**
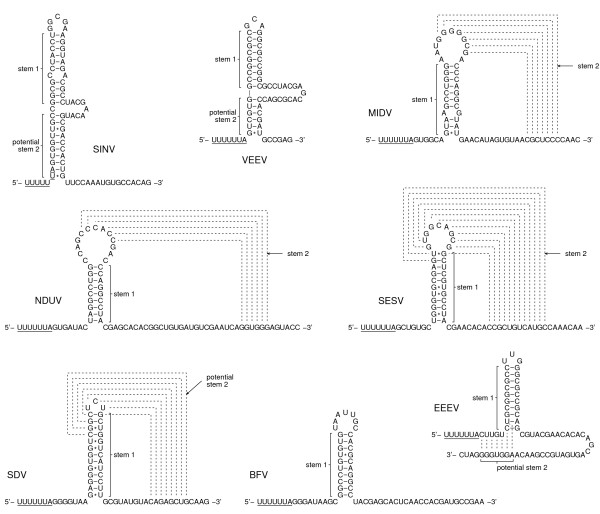
**Potential stimulatory RNA secondary structures for -1 frameshifting in representative alphavirus species**. Stems marked as 'potential' were *not *supported by dual luciferase mutational analyses (B Chung et al, in preparation), though it is possible that they may still be important in the context of the full 26S sgRNA in virus-infected cells. Viruses: Seal louse (SESV) – [GenBank:AF315122]; Middelburg (MIDV) – [GenBank:AF339486]; Venezuelan equine encephalitis (VEEV) – [GenBank:NC_001449]; Ndumu (NDUV) – [GenBank:AF339487]; Sindbis (SINV) – [GenBank:NC_001547]; Barmah Forest (BFV) – [GenBank:NC_001786]; Sleeping disease (SDV) – [GenBank:NC_003433]; Eastern equine encephalitis (EEEV) – [GenBank:NC_003899].

**Figure 3:**
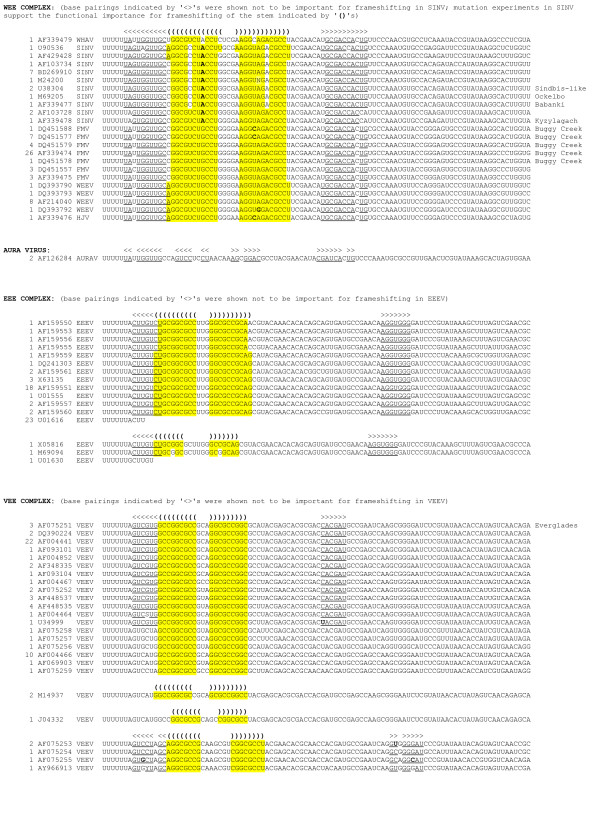
**Potential downstream RNA secondary structures in all sequences analysed. (Continued in Figure 4.)** As of 20 April 2008, there were 357 alphavirus sequences in GenBank with coverage of the U UUU UUA motif in the 6K cistron. The 100 nt region starting from the U UUU UUA motif, and including the first 93 nt of 3'-adjacent sequence, was extracted from all 357 sequences (although in 26 sequences a shorter region had to be used due to incomplete sequence data). Shown here are the 108 unique ≤100-nt sequences, plus an additional seven duplicate sequences also included since they have different species/strain annotations. The total number of duplicate sequences represented by each sequence shown is given in column 1, while column 2 gives an example GenBank accession number for the sequence, and column 3 gives the virus name abbreviation. Potential RNA secondary structures were identified using a combination of RNAfold and alidot [36], pknots [37], and manual inspection. Bases within potential stems are indicated either in colour or with underlines (if overlapping other potential stems) and potential base-pairings are indicated with brackets – '()', '[]' or '<>'. '<>' signify more dubious base-pairings, including stems that were experimentally shown not to affect frameshifting efficiency (dual luciferase assays with inserts comprising the U UUU UUA motif and 3'-adjacent sequence; B Chung et al, in preparation). Base variations that maintain base-pairings are marked in bold. Note that not all sequences in GenBank represent functional (infectious) viruses and it is possible that certain sequences whose shift site and/or predicted RNA structure do not conform with the majority of isolates for the same species may represent defective viruses – for example the non-standard slippery heptanucleotide in the SPDV sequence AJ012631 is due to a 36-codon deletion in 6K relative to other SPDV sequences.

**Figure 4:**
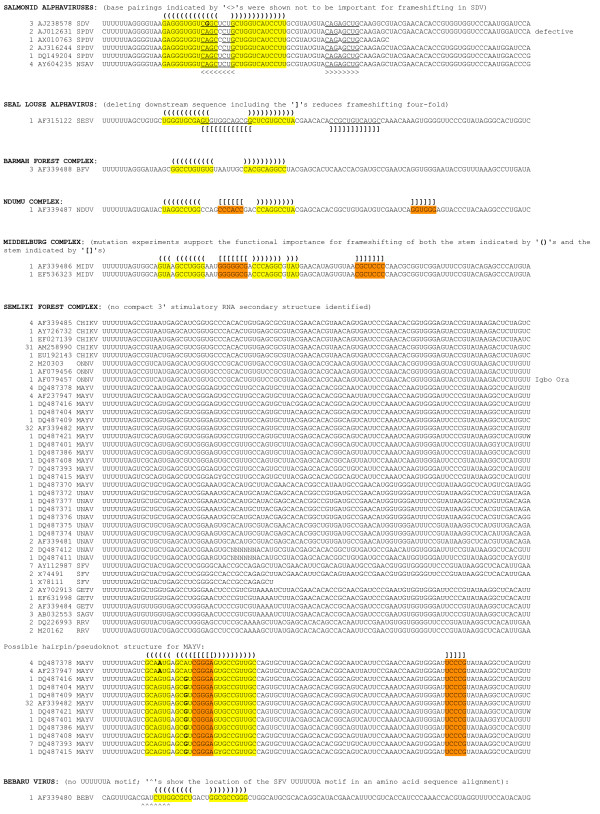
Potential downstream RNA secondary structures in all sequences analysed. (Continued from Figure 3.)

Of the 328 sequences that contain ≥90 nt 3' of U UUU UUA, potential 3' RNA secondary structures (Figures 2, 3, 4) were found in all except, possibly, Aura virus and the SF complex. In some species the structure is exceptionally stable – e.g. in VEEV there is a hairpin stem comprising nine consecutive GC-pairs, while the salmonid alphaviruses have a predicted stem of 13 nt. The predicted hairpin stem in the WEE complex is additionally supported by compensatory mutations (paired mutations that preserve the base-pairings) – e.g. one position in the stem is occupied by an A:U, G:C or G:U pair depending on the species and strain (Figure 3). Other species – such as MIDV, SESV and Ndumu virus – have potential pseudoknots.

**Figure 5:**
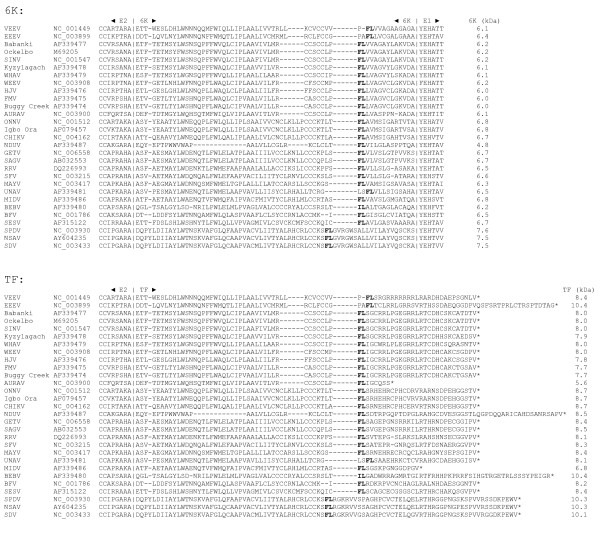
**Peptide sequences for the 6K and TF proteins for representative alphavirus sequences**. The frameshift site (amino acids 'FL', except in BEBV) is shown in bold. For BEBV, which lacks the U UUU UUA motif, the approximate location of the presumed frameshift was determined by alignment to the other sequences. '|'s represent the E2-6K and 6K-E1 cleavage sites and '*'s represent the TF protein termination codon.

**Figure 6:**
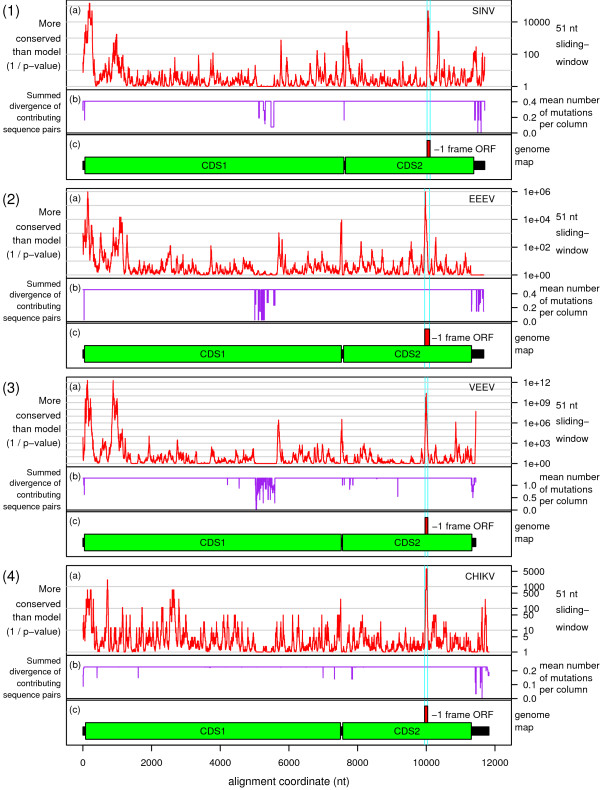
**Phylogenetic nucleotide conservation plots for selected alphavirus within-species full-genome sequence alignments**. The nucleotide conservation in a 51-nt sliding window is expressed as a *p*-value plot, giving the probability that the conservation in the window would be as great or greater than that observed, if a given null model (CDS annotation) was true. Here the null model was set to 'non-coding' in order to give a straightforward nucleotide conservation plot. Plots are given for alignments of **(1a) **7 Sindbis virus (SINV) sequences, **(2a) **9 Eastern equine encephalitis virus (EEEV) sequences, **(3a) **22 Venezuelan equine encephalitis virus (VEEV) sequences, and **(4a) **19 Chikungunya virus (CHIKV) sequences. Panels **(1-4b) **show the phylogenetically summed sequence divergence (mean number of base variations per nucleotide column) for the sequences that contribute to the statistics at each position in the alignment. In any particular column, some sequences may be omitted from the statistical calculations due to alignment gaps. Statistics in regions with lower summed divergence (i.e. partially gapped regions) have a lower signal-to-noise ratio and/or may be omitted from the plot. Panels **(1-4c) **show the location of the non-structural (CDS1; green) and structural (CDS2; green) CDSs, the non-coding regions (black), and the location of the overlapping -1 frame ORF (red), in the GenBank RefSeqs NC_001547 (SINV), NC_003899 (EEEV), NC_001449 (VEEV) and NC_004162 (CHIKV). The location of the U UUU UUA motif coincides with the 5' end of this ORF. Plots were produced with the CDS-plotcon webserver (Firth, unpublished).

The downstream -1 frame ORF is short (generally 26–31 codons, though as short as 8 codons in Aura virus, and reaching 50 codons in Ndumu virus) resulting, after presumed cleavage at the N-terminus of 6K, in the alternative protein TF (Figure 5). The N-terminal end of TF retains ~71–83% of 6K – including the hydrophobic transmembrane region [12] – but has an altered and generally elongated C-terminal end (typically ~8 kDa product), often with even more Cys residues than 6K (Figure 5). This region of the genome shows unusually high nucleotide conservation (Figure 6) – as expected for sequence that is coding in two overlapping reading frames, besides containing the frameshift stimulatory signals and the 6K-E1 cleavage site.

Of the four sequences (out of 357) that do not contain the U UUU UUA motif, two are identical defective Salmon pancreas disease virus sequences with C UUU UUA as a direct consequence of a 36-codon deletion (between the 'C' and first 'U') within 6K [25] (11 other salmonid alphavirus sequences all have U UUU UUA). Another – an EEEV sequence with U UUU UUG – may also represent a defective sequence since there are 59 other EEEV sequences all with U UUU UUA. The fourth sequence – the only 6K sequence for Bebaru virus – appears to completely lack the U UUU UUA motif. However, Bebaru virus does contain a 47-codon -1 frame ORF (5' terminus determined by alignment to the frameshift site in other alphavirus species), or up to 94 codons (if frameshifting occurs at a different location), suggesting that TF is also present in Bebaru virus.

### Amino acid sequencing confirms expression of the predicted transframe protein TF

**Figure 7:**
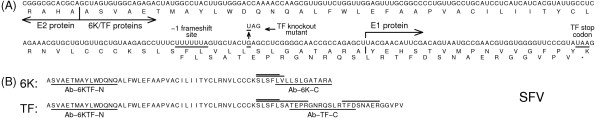
**Nucleotide and amino acid sequences for 6K and TF in SFV**. (A) Nucleotide sequence for 6K and flanking regions, with the polyprotein and -1 frame amino acid sequences given below. The cleavage sites between E1, E2 and 6K are marked. Also marked are the frameshift site U UUU UUA, the TF termination codon, and the position of the point mutation used for the knockout mutant TF^-^. (B) Amino acid sequences for the 6K and TF proteins. Three antigens against which three separate Abs were raised are marked by underscores. Peptides with clear mass spectrometry detections are marked by overscores.

Liquid chromatography tandem mass spectrometry (LC/MS/MS) of in-gel trypsin and chymotrypsin digests of low molecular mass products from purified SFV virions demonstrated the presence of a number of tryptic peptides that derive from the C-terminal (frameshifted) region of TF and that are not present in the non-frameshifted 6K protein or in any other SFV protein (Table 1; Additional file 1; MASCOT scores ≥ 20; mass errors < 3 ppm). These peptides include SLSFLSATEPR and TFDSNAER (Figure 7B). Presence of the peptide SLSFLSATEPR, whose coding sequence spans the frameshift site U UUU UUA, indicates that tandem slippage occurs (i.e. A-site tRNA^Leu ^pairs to UUA and then slips to UUU, while P-site tRNA^Phe ^slips on the tetranucleotide U UUU). The slippage site-encoded peptides SLSFL and SLSFLV were also detected. Interestingly the latter, due to the C-terminal 'V', could only originate from the non-frameshift 6K protein, though relative amounts could not be established from this data. Additionally, various subsequences of the peptides MLEDNVDRPGYYDLLQAALTCR and ENNAEATLR – which derive from the E3 protein – were also detected. The mass spectrometry data also supported assignment of the trans-slippage site peptide SLSFF (Table 1; Additional file 1; MASCOT score = 15). This indicates the presence of some P-site slippage – i.e. P-site tRNA^Phe ^slips on the tetranucleotide U UUU with no tRNA in the A-site, and then a new tRNA^Phe ^pairs to UUU in the A-site.

**Table 1:** Mass spectrometry MASCOT peptide identifications

Origin	Peptide	Observed	Mr(expt)	Mr(calc)	Delta	ppm	Score	Expect
6K/TF	K.SLSFL.S	566.3198	565.3125	565.3111	0.0013	2.30	24	6.5e-4
6K	K.SLSFLV.L	665.3886	664.3813	664.3795	0.0017	2.56	27	1.1e-4
TF?	K.SLSFF.S	600.3032	599.2959	599.2955	0.0005	0.83	15	0.0034
TF	K.SLSFLSATEPR.G	604.3198	1206.6250	1206.6244	0.0006	0.50	61	4.3e-8
TF	L.SATEPR.G	660.3338	659.3265	659.3238	0.0027	4.10	11	0.0089
TF	R.TFDSNAER.G	470.2130	938.4115	938.4093	0.0021	2.24	55	5.8e-7
TF	R.GGVPV.-	428.2513	427.2441	427.2430	0.0010	2.34	16	0.0062
E3	Y.DLLQAAL.T	743.4319	742.4246	742.4225	0.0021	2.83	32	3.2e-5
E3	L.EDNVDRPGYY.D	1227.5310	1226.5237	1226.5203	0.0034	2.77	32	1.3e-4
E3	R.MLEDNVDRPGYY.D + Oxidation (M)	744.3299	1486.6452	1486.6398	0.0054	3.63	58	8.1e-8
E3	R.MLEDNVDRPGYYDLLQ.A + Oxidation (M)	978.9579	1955.9013	1955.8934	0.0078	3.99	39	1.2e-5
E3	R.MLEDNVDRPGYYDLLQA.A + Oxidation (M)	1014.4784	2026.9423	2026.9306	0.0117	5.77	22	0.0013
E3	R.MLEDNVDRPGYYDLLQAAL.T + Oxidation (M)	1106.5379	2211.0613	2211.0517	0.0095	4.30	27	2.9e-4
E3	R.MLEDNVDRPGYYDLLQAALT.C + Oxidation (M)	771.7088	2312.1046	2312.0994	0.0052	2.25	67	7.2e-8
E3	R.MLEDNVDRPGYYDLLQAALTCR.N + Oxidation (M)	858.0809	2571.2208	2571.2097	0.0111	4.32	27	2.9e-4
E3	Y.ENNAEATLR.M	509.2524	1016.4903	1016.4886	0.0016	1.57	62	3.4e-8

No purely N-terminal 6K/TF peptides were detected. The predicted tryptic cleavage products for this region are ASVAETMAYLWDQNQALFWLEFAAPVA**C**ILIITY**C**LR and NVL**CCC**K, both of which contain potential palmitoylation sites (Cys residues; [15,16]). Although the various possibilities for palmitoylation were taken into account in the peptide database search, poor ionization of peptides with palmitoyl derivatives could explain why there were no detections. Furthermore, large peptides such as the 37-mer are unlikely to trigger the MS/MS scan.

### Phenotype of a TF knockout/truncation mutant (TF^-^)

To investigate the phenotype of a TF knockout mutant, we introduced a point mutation into an infectious clone of SFV. The mutant, TF^-^, differs from wild-type (WT) SFV by just a single point mutation, CUG → CUU, 9 nt 3' of U UUU UUA (polyprotein-frame codons shown). The mutation is synonymous with respect to the polyprotein frame, but introduces a premature termination codon (UAG) into TF (Figure 7A). Phenotypes were assessed by plaque assays in BHK cells. The TF^- ^mutant showed only an ~56% reduction in growth (7.5 ± 0.4 × 10^8 ^PFU/ml) relative to WT (1.7 ± 0.1 × 10^9 ^PFU/ml). RT-PCR and sequencing of RNA extracted from the infected cells used to propagate virions for the plaque assays, as well as a portion of the virions, confirmed the presence of the appropriate virus (WT or TF^-^; data not shown). Note that codon usage *may *be a factor in the reduced-growth phenotype of TF^-^, since the CUU codon is used ~5× less frequently in the SFV genome than the CUG codon (20 and 102 occurrences, respectively).

### Location and abundance of TF

**Figure 8:**
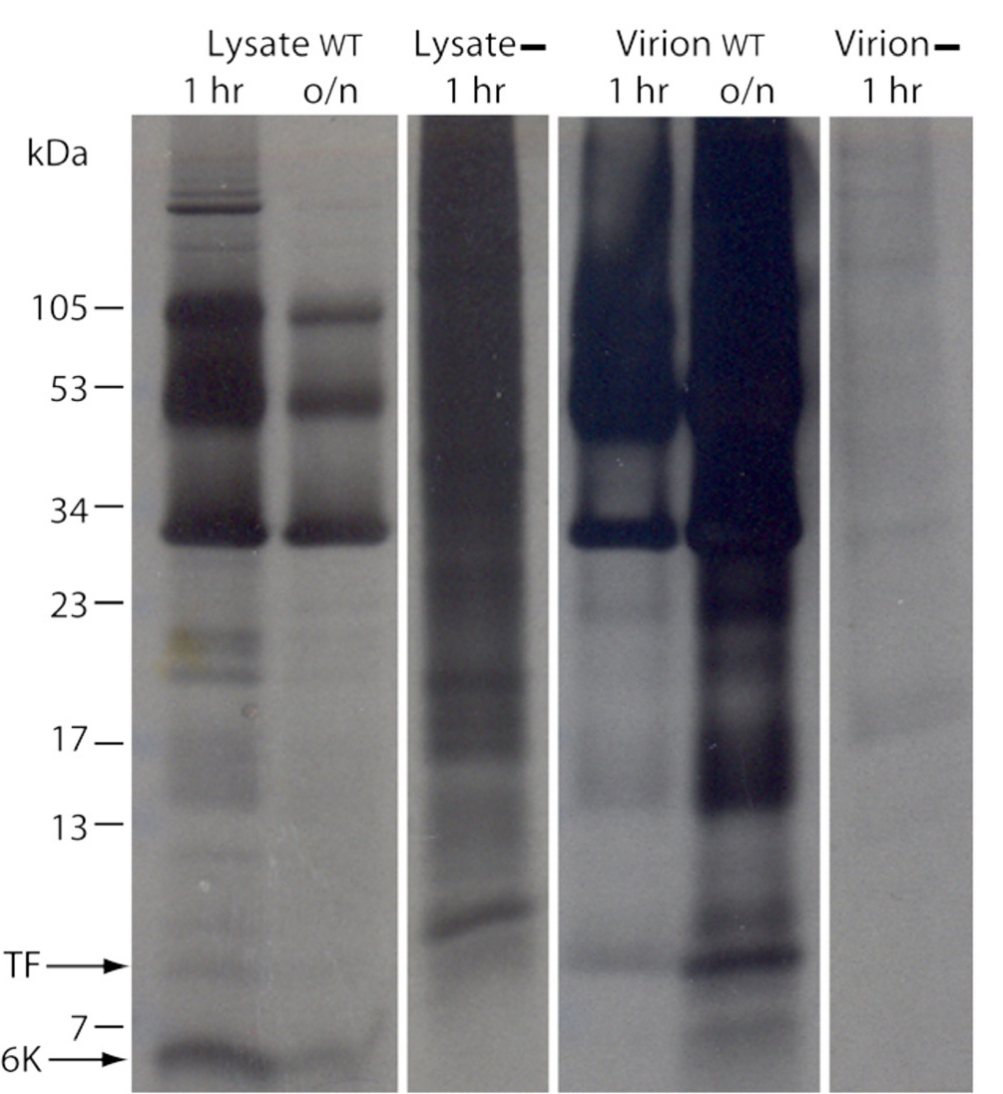
**Virus-specific detection of SFV 6K and TF proteins**. Lanes 1–3: total lysate from SFV-infected (WT; 1 hr and overnight, o/n) and non-infected (-) cells. Lanes 4–6: virions purified from the media (WT) and mock purified virions from non-infected cells (-). Equal amounts of transfecting RNA and cells were used for each sample. All lanes are from the same gel – exposed on x-ray film for 2 weeks to enhance the faint bands corresponding to any low molecular mass products.

**Figure 9:**
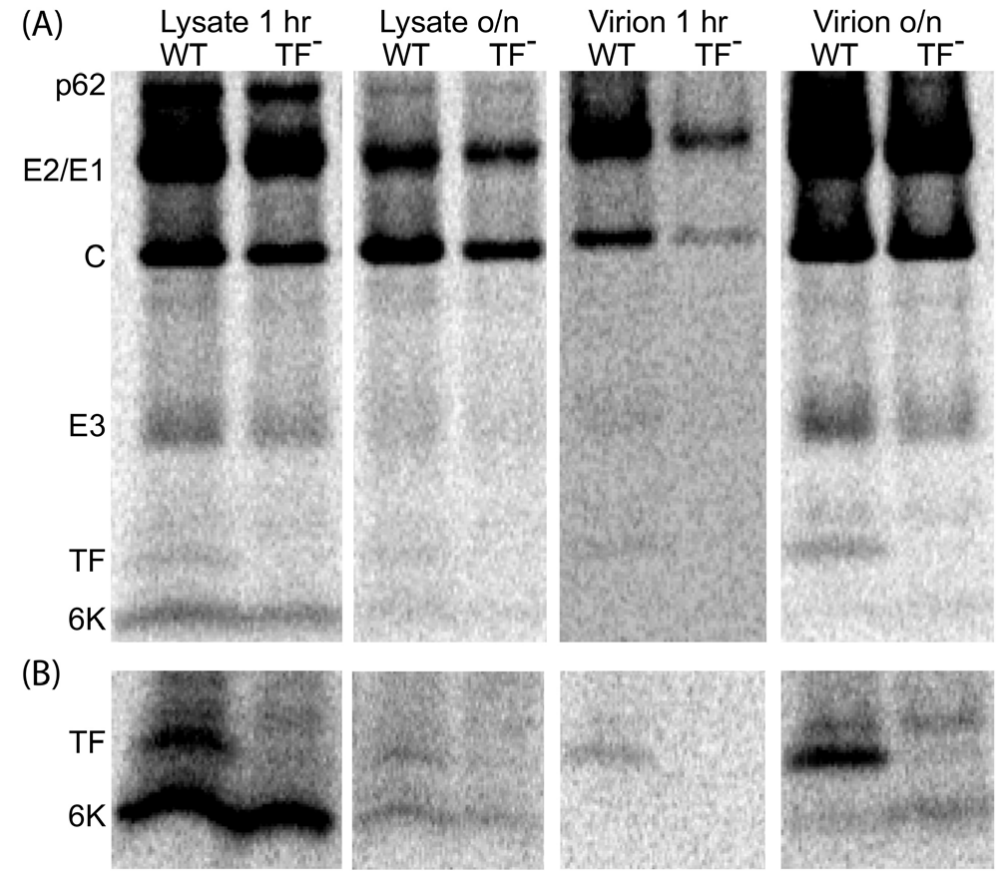
**Detection of SFV 6K and/or TF proteins for WT and TF^- ^viruses**. (A) SFV-infected cells were labelled with [^35^S]Met/Cys and cell lysates (1 hr and overnight, o/n) and purified virions were analyzed by SDS-PAGE. Equal amounts of transfecting RNA and cells were used for each sample. Lanes 1–4 and 7–8 are from the same gel, lanes 5–6 are from a separate gel; Phospho-Imager, 2 days exposure. Negative controls are shown in Figure 8. (B) As above, but with higher sample loading.

SFV-infected cells were labelled with [^35^S]Met/Cys, and proteins from cell lysate and from purified virions were subjected to SDS-PAGE. Consistent with previous results (e.g. [7]; SINV), a virus-specific 6K doublet was observed (Figure 8), where the more slowly migrating band (consistent with the predicted size of TF, ~8.3 kDa) was much fainter than the other band (consistent with the predicted size of 6K, ~6.6 kDa) for cell lysate (Figure 9, lanes 1, 3), but was the predominant band for the virion sample (Figure 9, lanes 5, 7). Correspondence of the more slowly migrating band to TF was verified by comparing migration patterns for WT SFV and the TF^- ^mutant on the same SDS-PAGE. In the TF^- ^lysate, the more slowly migrating band disappeared, while the intensity of the other band remained essentially unchanged (Figure 9, lanes 2, 4), thus conclusively demonstrating that the more slowly migrating band corresponds to TF. Interestingly, there *may *be a very small amount of TF in the TF^- ^virion sample (Figure 9, lane 8), indicating some reversion from TF^- ^to WT. A fainter band migrating just behind the TF band (e.g. Figure 9, lanes 7, 8) may represent some unglycosylated E3 (glycosylated E3 migrates at ~13 kDa; the predicted size of unmodified E3 is ~7.4 kDa).

**Figure 10:**
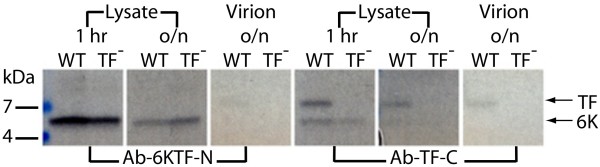
**Immunoprecipitation of SFV 6K and/or TF proteins for WT and TF^- ^viruses**. SFV-infected cells were labelled with [^35^S]Met/Cys and cell lysates (1 hr and overnight, o/n) and purified virions were subjected to immunoprecipitation with Ab-6KTF-N or Ab-TF-C followed by SDS-PAGE, and exposed on x-ray film for 3 weeks. Equal amounts of transfecting RNA and cells were used for each sample.

Although comparison of the WT and TF^- ^SDS-PAGE migration patterns conclusively identifies the TF band, further confirmation for both the 6K and TF bands was obtained via immunoprecipitation using separate Abs raised against two 14 amino acid peptides (Figure 7B) – Ab-6KTF-N (SFV 6K/TF amino acids 2–15; N-term) and Ab-TF-C (SFV TF amino acids 52–65; C-term). A third Ab, Ab-6K-C, raised against SFV 6K amino acids 49–60 (6K C-term) was also produced, but proved ineffective due to the poor antigenicity of this peptide. In fact, the very small size and overall poor antigenicity of the 6K protein proved very restrictive, so that the Ab-6KTF-N antigen was also predicted to be quite poor. In lysate from SFV-infected cells, Ab-TF-C preferentially immunoprecipitated TF (Figure 10, lanes 7, 9). A small amount of 6K also visible in lane 7 is presumably a result of imperfect purification in the immunoprecipitation – indeed, this occurred to some extent in all lanes for the higher mass, higher Met/Cys-content, virus proteins (data not shown). Nonetheless, given that TF is much less abundant than 6K in the non-immunoprecipitated cell lysate (Figure 9, lane 1), the affinity of Ab-TF-C for TF is clear. Ab-TF-C also immunoprecipitated TF from purified SFV virions (Figure 10, lane 11). Ab-6KTF-N, on the other hand, preferentially immunoprecipitated 6K from cell lysate (Figure 10, lanes 1, 3). Although Ab-6KTF-N was expected to also immunoprecipitate TF, this was not observed (except for a very faint band in the virion sample; Figure 10, lane 5) – perhaps partly due to the much lower abundance of TF relative to 6K in cell lysate, but another possibility is that the high degree of palmitoylation inferred for TF, but not 6K (see Appendix 1), interferes with Ab binding. As expected, in lysate from cells infected with the TF^- ^mutant, Ab-6KTF-N immunoprecipitated 6K (Figure 10, lanes 2, 4), but Ab-TF-C failed to immunoprecipitate TF (Figure 10, lanes 8, 10). Similarly, Ab-TF-C failed to immunoprecipitate TF from TF^- ^virions (Figure 10, lane 12).

In SFV, both 6K and TF have one Met and five Cys residues, so the 6K:TF molar ratio is proportional to the ratio of the 6K and TF band intensities. In cell lysate, the molar amount of TF relative to 6K, as determined by densitometry, was ~18% (Figure 9, lane 1), implying a frameshift efficiency, TF/(6K+TF), of ~15%. The molar ratio of 6K+TF to the capsid protein (8 Met, 4 Cys) was close to the expected value of unity. In contrast, in protein prepared from purified virions, the molar ratio of TF may have been as high as ~15% relative to the capsid protein, but only very small amounts of 6K (< 25% the amount of TF) were detected (Figure 9, lane 7). Interestingly, when TF was knocked out, the amount of 6K in the virion sample seemed to increase (Figure 9, lane 8); though an alternative explanation is that this band now represents palmitoylated C-terminally truncated TF (cf. discussion).

**Figure 11:**
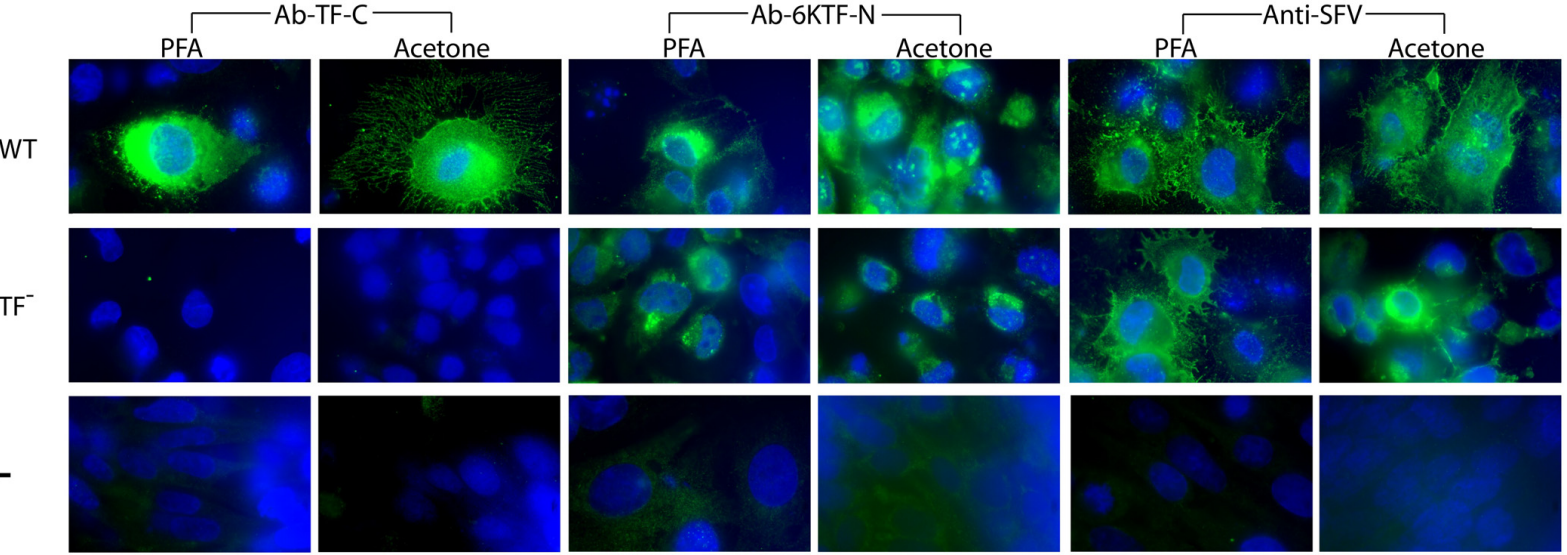
**Immunofluorescence of SFV-infected cells showing location of 6K and TF proteins**. Green fluorescence indicates Abs binding to target peptides. Cell nuclei are stained blue. Cells fixed in acetone are permeabilized, allowing intracellular Ab staining. Cells fixed in 4% PFA are not permeabilized, thus only allowing Abs to bind to peptides at the cell surface. Cells were infected with WT SFV4 virus (WT), the TF knockout mutant (TF^-^), or are non-infected controls (-). TF^- ^serves as an additional control for Ab-TF-C. See also Additional file 2.

Immunofluorescence imaging of both permeabilized and non-permeabilized SFV-infected cells resulted in strong fluorescence when using Ab-TF-C, indicating that TF is present both intracellularly and at the cell surface (Figure 11, Additional file 2A). No significant fluorescence was detected, when using Ab-TF-C, for non-infected cells or, importantly, for cells infected with the TF^- ^mutant (confirming the specificity of Ab-TF-C). In images produced using Ab-6KTF-N, fluorescence was generally weaker (presumably reflecting the poorer antigenicity of the N-terminal peptide) and there was some background fluorescence for non-infected cells. Nonetheless there was clear above-background fluorescence for both WT- and TF^-^-infected cells, showing that 6K is also present both intracellularly and at the cell surface (Figure 11, Additional file 2B). A general anti-SFV Ab showed strong fluorescence for both WT- and TF^-^-infected cells, but no significant fluorescence for non-infected cells (Figure 11, Additional file 2C).

### Analysis of 3' elements that stimulate frameshifting

The frameshift efficiencies of the slippery heptanucleotide U UUU UUA and 3'-adjacent sequence from SFV, SINV, EEEV, VEEV, MIDV, SDV and SESV were compared in cell culture by means of dual luciferase reporter assays [26]. A complete description of this work is presented in a separate manuscript (B Chung et al, in preparation) but certain results are summarized here as they are pertinent to the discussion that follows. Frameshift efficiencies ranging from 5% to 40% (depending on species) were measured for WT sequence, with results for SFV and SINV ranging from 10% to 17% (depending on insert length). The close agreement with our measurement of ~15% for SFV-infected cells (see above), and the range of 10–18% that may be derived from data presented in refs. [7,15] for SINV-infected cells (see Appendix 1), lends credence to the supposition that these values are not unrepresentative of the frameshift efficiencies in the context of the full 26S sgRNA in virus-infected cells. In any case, it is clear that the U UUU UUA motif and 3'-adjacent sequence are capable of stimulating high levels of frameshifting. In contrast, a SINV insert in which the slippery heptanucleotide U UUU UUA was mutated to U UUC UUA had <0.5% frameshifting. Additional constructs in which groups of nucleotides were mutated to disrupt predicted 3' stimulatory structures and/or to maintain predicted structures but with reversed base-pairings, supported the predicted hairpin stem in SINV and a pseudoknot in MIDV (Figures 2, 3, 4; B Chung et al, in preparation). Comparison of deletion series of inserts indicated that only a single stem was important in SINV, EEEV, VEEV and SDV but, in SESV, a predicted pseudoknot was supported. Interestingly, we were unable to find a compact 3' RNA secondary structure in the SF complex (with the possible exception of Mayaro virus), though the dual luciferase assays did show that the 3' sequence – in particular the first 14 nt after the U UUU UUA motif – is important for efficient frameshifting.

## Discussion

### Previous analyses of the 6K doublet

In light of the results presented here, it is vitally important to revisit and reinterpret many earlier results regarding the 6K doublet. The fact that '6K' is present in two forms was first demonstrated and investigated by Gaedigk-Nitschko & Schlesinger [7]. The authors concluded that one form, which they labelled '4K', was a partially acylated form of the other, which they labelled '6K'. We now propose that '4K' equates to 6K and '6K' is in fact TF, both of which may be acylated to varying degrees. A full reanalysis of the diverse results presented in refs. [7,15,16] is given in Appendix 1. Key observations include: (*i*) a number of anomalies in the old data that were inconsistent with the old explanation for the 6K doublet, are perfectly consistent with the new frameshifting explanation; (*ii*) after adjusting for the SINV TF:6K Cys ratio being 9:5, the frameshifting efficiency in SINV-infected cells may be calculated from the old data, and ranges from 10–18%; (*iii*) TF appears to be much more heavily palmitoylated than 6K (~1 fatty acid on 6K and ~5–7 on TF for SINV; fewer fatty acids on TF for SFV); and (*iv*) in SINV and SFV, TF but not 6K is present in the virion.

### Involvement of 6K and TF in virus budding

Previous studies of 6K mutants have demonstrated a number of roles for the 6K region. There is, at some level, a dichotomy of phenotypes. For 6K deletion mutants, Δ6K, (which produce neither 6K nor TF), virus yield tends to be greatly reduced, but those virions that are produced appear to have normal morphology and infectivity [17,19] (SFV). In this case, the C-term of E2 replaces the C-term of 6K as the signal peptide for E1 so that the envelope proteins E1 and E2 are processed more-or-less normally [6,8,17]. Other mutants which appear to knock out 6K/TF function via partial deletions [21] (SINV), insertions [18] (SINV), or a SINV-RRV 6K chimera that disrupts the 3' end of the TF sequence [10], have a similar phenotype, although envelope protein processing may also be defective. On the other hand, when just one or a few amino acids within 6K/TF are mutated to different amino acids, the yield is reduced but often the virion morphology is also affected, with many virions appearing distorted and multicored (i.e. comprising several nucleocapsids within one membrane structure) [15,16,20,27]. Nonetheless, virions produced by such mutants are often still infectious. Thus it has been proposed that a major role of 6K lies in late stage virus assembly or budding, though it is now unclear whether this role is played by 6K or TF or both.

The 6K protein itself has been shown to possess properties typical of a viroporin (i.e. small viral proteins that, among other functions, increase membrane permeability and create conditions that favour virus budding; reviewed in [13]). Besides its hydrophobic transmembrane region, individual expression of 6K increases membrane permeability in *E. coli *[28] (SFV), mammalian cells [12] (SINV), and *Xenopus *oocytes [14] (SINV). (Note, however, that some of these results are now confounded, since there *may *be co-expression of low levels of a C-terminally truncated [6K-sized] TF product, whose phenotypic effects may be mixed with those of 6K.) RRV and Barmah Forest virus 6K proteins have been shown to form ion channels in planar lipid bilayers [11]. Furthermore, most or all of 6K appears to associate with p62-E1 (p62 is the precursor of E3 and E2) heterodimers soon after synthesis, with which it is transported to the cell surface [9] (SFV). Thus it appears likely that 6K, at least, is involved in budding.

It is interesting however that, while 6K and TF both share the transmembrane region, in TF but not in 6K this tends (depending on species) to be followed by a region rich in basic residues – a characteristic of HIV-1 Vpu and several other viroporins [13]. Indeed SINV 6K partial deletion mutants have been shown to be partially complemented by Vpu [22] – a result which could reflect functions of either 6K or TF. Thus TF may also play an important role in budding. Frameshifting may be necessary to provide TF with a hydrophilic C-term while maintaining a hydrophobic C-term in 6K to act as the signal peptide sequence for E1. The heavy palmitoylation inferred for TF (see Appendix 1) suggests an association with lipid bilayers, particularly the plasma membrane. (It remains to be determined what directs the much heavier palmitoylation inferred for TF than for 6K – for example, whether it is related to the differing C-terminal sequences, or to the fact that 6K, but not TF, is synthesized C-terminally joined to E1. In any case, the difference is likely to have important consequences for the differential sorting, function and stability of the two proteins [29].) Finally, our immunofluorescence data also suggest that TF plays a role at the cell surface.

Mutation of three potential Cys palmitoylation sites (all 5' of the frameshift site) in SINV 6K/TF resulted, as expected, in reduced palmitoylation of '6K' (i.e. TF), and a phenotype comprising virions that were infectious but often distorted and multicored, slower budding, and yield reduced to 10–30% of WT [15]. A similar phenotype was observed for a mutant in which four Cys residues were replaced [16]. Since it appears to be TF that is heavily palmitoylated, rather than 6K, these phenotypes may relate more to the function of TF than 6K (although impairment of a 6K function due simply to the altered 6K peptide sequence, and/or removal of the low amount of 6K palmitoylation, can not be ruled out). In other words, the effects on virion morphology and/or budding usually associated with 6K, *may *in fact be largely due to TF. This is also consistent with the phenotype of the SINV-RRV chimera of ref. [10] – instead of reduced budding being due to the replacement of SINV 6K with RRV 6K, it could be due to the absence of both SINV and RRV WT TF.

Further evidence comes from complementation studies using a SINV mutant in which amino acids 24–45 of 6K had been deleted [21]. The deletion removes the frameshift site so that no TF can be produced. The mutant was defective in the processing and transport of envelope proteins (as expected since the C-term of 6K contains the signal peptide for E1) and in plaque phenotype. A revertant virus, containing a point mutation in the deleted 6K gene (which increased hydrophobicity), corrected the defects of envelope protein processing and transport (presumably by partially restoring the signal peptide for E1), but it still remained attenuated compared to WT, exhibiting defects in virus budding. Neither mutant nor revertant viruses were complemented by the co-expression *in trans *of a WT 6K gene. That the mutant was not complemented *in trans *is not surprising, since the E1 signal peptide clearly can not operate *in trans*. However, the fact that the revertant also retained defects in virus budding when 6K was co-expressed *in trans*, is strong evidence that TF plays an important role in budding (in SINV, the TF coding sequence extends ~15 codons 3' of the 6K coding sequence and, therefore, TF will not be present in its native form when 6K is expressed *in trans*).

On the other hand, our own SFV TF knockout mutant, TF^-^, only exhibited an ~56% reduction in growth compared to WT. While statistically significant, and consistent with a role for TF in virus yield, the reduction is nonetheless modest and does not account for the full reduction in growth seen for the Δ6K mutant (50–98%; [17,19]), unless the difference is due to reversion of TF^- ^to WT. Thus we propose that probably *both *6K and TF play roles in virus budding. Alternatively, perhaps it is simply the absence of the C-term of 6K, and/or the fact that TF is not synthesized C-terminally joined to E1, that is important for TF function. These properties are preserved in the truncated TF protein produced by the TF^- ^mutant, and this may explain the fairly modest effect on virus growth. A full characterization of the newly discovered TF protein is beyond the scope of this paper, and will be addressed in future work.

### A possible role for TF in the virion

Our SDS-PAGE results show that it is predominantly TF rather than 6K that is incorporated into the SFV virion. These results are supported by our reinterpretion of the SINV data in refs. [7,15] (see Appendix 1). The abundance of '6K' (i.e. TF) in the virion has been previously determined as ranging from 7–30 copies [1], cf. 240 copies each for the capsid and envelope proteins. However previous estimates now need to be adjusted in cases where the number of Met or Cys residues (as appropriate) in the TF sequence differs from the number in the assumed 6K sequence. Table 1 of ref. [7] shows, after multiplying by 5/9 (for the re-evaluated number of Cys residues), that the molar ratio of TF in the SINV virion is ~4.4% relative to the other virion components. Elsewhere in ref. [7], corrected estimates range from 4.4% to 6.7%. Similarly, the ranges 25–30 [18] and 24–30 [20] for SINV translate to a range of 5.6% to 6.9%. The value of ~3% derived in ref. [9] for the SFV virion using [^35^S]Met remains unchanged (here both TF and 6K have one Met residue). Intriguingly, the TF band in our SDS-PAGE results appeared darker than expected for a molar ratio of 3–7%, and densitometry indicated that the molar ratio may have been as high as ~15%.

As numerous authors have previously noted with regards to 6K [15,18,19,23,27], it is unclear whether it is the presence of TF within the virion – as a structural component – that is responsible for the virion defects seen in 6K/TF mutants, or whether 6K/TF play their role solely at the budding and assembly stage – i.e. 6K/TF are required to achieve proper budding resulting in a 'stable' virion but the fact that TF is incorporated into the virion is accidental, and possibly restricted only to defective virions.

Given that the discrepancy between the molar ratio of TF in the virion (~3–15%) and the molar ratio of TF at translation (~10–18%) is considerably less than previously supposed (i.e. for 6K), the argument for '6K' (i.e. TF) being merely an accidental inclusion in the virion is now perhaps weakened. Apparently, when WT TF or no TF is incorporated into the virion, virion morphology is more-or-less normal [17-19,21], but when mutant TF is incorporated into the virion, virions may be distorted [15,20]. This could be a direct effect of TF as a structural component of the virion (though an alternative model, proposed by ref. [18], is that when 6K/TF are absent, a low rate of budding of normal virions takes place at regions where the cell membranes naturally have high curvature and thus does not require the membrane-altering properties of 6K/TF). The observation that the aberrant virion morphology of some SINV 6K/TF mutants is restored to WT by certain mutations in E2 [20,27] may also support a structural role for TF. If TF is indeed a symmetrically-arranged structural component of the virion, then the fact that the location of '6K' in the virion has not yet been determined by cryo-electron microscopy [30] may partly be a consequence of attempting to fit 6K instead of TF into the cryo-EM maps. Since Δ6K virions appear to have normal morphology and infectivity, if TF does play a role in the virion, then it is presumably a relatively minor role and/or only important under certain conditions. Nonetheless, compared to WT, SFV Δ6K virions have been shown to exhibit reduced stability, as manifested by a greater sensitivity to inactivation by heat [19] and low pH [23], besides decreased fusion capability [23].

The frameshift efficiency may be tuned to help control the stoichiometry of TF in the virion, although the fact that the level of frameshifting is apparently substantially higher than the ~5% virion molar ratio generally found for TF, and potentially varies between species (B Chung et al, in preparation), hints that varying amounts of TF may be 'diverted' *en route *to the developing virion and/or that TF also plays other roles in infected cells, such as in budding and membrane permeabilization, as discussed above. Implicitly, production of E1 is predicted to be reduced by the level of frameshifting (i.e. ~10–18% for SFV and SINV), thus leaving a surplus of C, E3 and E2.

## Conclusion

We have demonstrated the existence of a ribosomal -1 frameshift site in the alphavirus structural polyprotein, that gives rise to the transframe protein TF, and demonstrated that it is primarily TF, rather than 6K, that is incorporated into the virion. We suggest that TF plays a stabilizing role in the virion structure, 6K plays a role in envelope protein processing, and probably both TF and 6K play a role in virus budding. The functional importance of frameshifting – as reflected by its wide conservation – may be to provide TF with a hydrophilic C-term while maintaining a hydrophobic C-term in 6K, and/or to help control the stoichiometry of TF in the virion.

Evidence presented here includes: (*i*) the nearly ubiquitous presence of a U UUU UUA motif in the 6K coding sequence throughout the *Alphavirus *genus; (*ii*) the presence of stable hairpin or pseudoknot RNA structures just 3' of the U UUU UUA motif in most alphavirus complexes; (*iii*) mass spectrometry of 6–8 kDa products isolated from purified SFV virions clearly shows the presence of the predicted transframe peptide sequences; (*iv*) a TF knockout mutant, that differed from WT SFV by a single point mutation synonymous in the polyprotein frame, showed an ~56% reduction in growth; (*v*) an SDS-PAGE of purified SFV virions and lysate from SFV-infected cells showed virus-specific bands at ~8.3 kDa (the predicted size of TF) and at ~6.6 kDa (the predicted size of 6K), with the ~8.3 kDa band being much fainter than the ~6.6 kDa band for cell lysate, but with the ~8.3 kDa band being much stronger than the ~6.6 kDa band for the virion; and (*vi*) identification of the ~6.6 kDa and ~8.3 kDa bands as corresponding to 6K and TF, respectively, was confirmed both by comparison of SDS-PAGE migration patterns for WT and TF^- ^mutant viruses and by immunoprecipitation. Furthermore, dual luciferase assays confirmed that the slippery heptanucleotide U UUU UUA and 3' sequence in SFV, SINV, EEEV, VEEV, MIDV, SDV and SESV were sufficient to induce high levels of ribosomal frameshifting in cell culture (B Chung et al, in preparation), while reinterpretation of diverse previous publications provides extensive additional evidence.

This discovery sheds new light on the enigmatic 6K protein. Previous investigations into the role of 6K were no doubt hampered by the false assumption that 6K and TF were one and the same protein and, consequently, the assumed amino acid sequence of TF was incorrect. As a virion component, knowledge of TF may be important for structural studies, and may have relevance to understanding virion stability, tropism, fusion and antigenicity, and hence the use of alphaviruses as gene therapy and vaccination vectors. The new results presented here have opened the way to a radical reinterpretation of existing data on the alphavirus 6K (and TF) proteins, and may allow more rapid future progress in their further characterization.

While the majority of known ribosomal frameshift sites provide access to a long out-of-frame ORF, here the out-of-frame ORF is unusual in that it has only 8–50 codons. Frameshifting involving such short ORFs is easy to overlook; indeed the only well-characterized cellular example – namely the eubacterial gene *dnaX *(reviewed in [31]) – was discovered fortuitously. Widespread use of ribosomal frameshifting into short out-of-frame ORFs has been proposed in *Saccharomyces cerevisiae *(and, by implication, other organisms) as a regulatory mechanism (e.g. by inducing nonsense-mediated mRNA decay) [32]. However, to what extent such sites are phylogenetically conserved remains to be addressed. The identification of a new phylogenetically conserved frameshift site in a genus as well-studied as the alphaviruses highlights the possibility that many such sites may remain undetected, not only in other viruses, but also in cellular genes.

From a bioinformatic point of view, the keys to efficiently locating such sites are (*i*) to search for phylogenetically conserved frameshift signals in order to find sites were the out-of-frame ORF is too short to detect with gene-finding software, and (*ii*) to search for short overlapping genes with sensitive gene-finding software in order to find sites where the frameshift signals do not conform to known patterns. We recently demonstrated (*ii*) by locating a short overlapping gene in the *Potyviridae *family, translated as a transframe fusion product by an as yet unidentified mechanism [33]. In this paper, we have demonstrated the efficacy of (*i*). Besides viral genomes, both methods are readily applicable to, for example, alignments of mammalian or vertebrate mRNAs.

## Methods

### Bioinformatics

As of 20 April 2008, GenBank contained whole-genome RefSeqs for 14 alphavirus species and 1643 alphavirus sequences in total (i.e. including partial sequences). Among these 1643 sequences, those with 6K coverage were identified by applying tblastn [34] using the 6K peptide sequences derived from the 14 RefSeqs as query sequences, resulting in 357 sequences.

### GenBank accession numbers for all sequences used

AF339480 AF126284 NC_003900 AF339477 AF339488 NC_001786 U73745 AF339474 DQ451559 DQ451560 DQ451561 DQ451562 DQ451563 DQ451567 DQ451568 DQ451569 DQ451570 DQ451571 DQ451572 DQ451573 DQ451574 DQ451575 DQ451576 DQ451577 DQ451578 DQ451579 DQ451580 DQ451581 DQ451582 DQ451583 DQ451584 DQ451585 DQ451586 DQ451587 DQ451588 DQ451589 DQ451590 DQ451591 DQ451592 DQ451593 DQ451594 DQ451595 DQ451596 DQ451597 DQ451598 DQ451599 AF339485 AF369024 AF490259 AM258990 AM258991 AM258992 AM258993 AM258994 AM258995 AY424803 AY726732 DQ443544 EF012359 EF027134 EF027135 EF027136 EF027137 EF027138 EF027139 EF027140 EF027141 EF210157 EF451142 EF451143 EF451144 EF451145 EF451146 EF451147 EF451148 EF451149 EF452493 EF452494 EU037962 EU192142 EU192143 EU244823 L37661 NC_004162 AF159550 AF159551 AF159552 AF159553 AF159554 AF159555 AF159556 AF159557 AF159558 AF159559 AF159560 AF159561 AY705240 AY705241 AY722102 CQ985850 DQ241303 DQ241304 EF151502 EF151503 EF568607 L20951 L37662 M69094 NC_003899 U01034 U01552 U01553 U01554 U01555 U01556 U01557 U01558 U01559 U01616 U01617 U01618 U01619 U01620 U01621 U01622 U01623 U01624 U01625 U01626 U01627 U01628 U01629 U01630 U01631 U01632 U01633 U01634 U01635 U01636 U01637 U01638 U01639 X05816 X63135 AF339475 DQ451557 DQ451558 DQ451564 DQ451565 DQ451566 AF339484 AY702913 EF631998 EF631999 NC_006558 AF339476 AF079457 AF339478 AF237947 AF339482 DQ001069 DQ487369 DQ487370 DQ487378 DQ487379 DQ487380 DQ487381 DQ487382 DQ487383 DQ487384 DQ487385 DQ487386 DQ487387 DQ487388 DQ487389 DQ487390 DQ487391 DQ487392 DQ487393 DQ487394 DQ487395 DQ487396 DQ487397 DQ487398 DQ487399 DQ487400 DQ487401 DQ487402 DQ487403 DQ487404 DQ487405 DQ487406 DQ487407 DQ487408 DQ487409 DQ487410 DQ487413 DQ487414 DQ487415 DQ487416 DQ487418 DQ487419 DQ487420 DQ487421 DQ487422 DQ487423 DQ487424 DQ487425 DQ487426 DQ487427 DQ487428 DQ487429 DQ487430 NC_003417 AF339486 EF536323 AF339487 AY604235 AY604236 AY604237 AY604238 M69205 AF079456 M20303 NC_001512 DQ226993 K00046 M20162 NC_001544 AB032553 AF339483 EF011023 AJ238578 AJ316246 NC_003433 AF315122 AY112987 BD317366 DQ189082 DQ189084 DQ189086 NC_003215 X04129 X74491 X78111 Z48163 U38304 U38305 AF103728 AF103734 AF429428 AY526355 BD269910 BD269911 CS227856 J02363 M24200 NC_001547 U90536 V00073 V01403 AJ012631 AJ316244 AX010750 AX010763 DQ149204 NC_003930 AF339481 DQ487371 DQ487372 DQ487373 DQ487374 DQ487375 DQ487376 DQ487377 DQ487411 DQ487412 DQ487417 AF004441 AF004458 AF004459 AF004464 AF004465 AF004466 AF004467 AF004468 AF004469 AF004470 AF004471 AF004472 AF004852 AF004853 AF069903 AF075251 AF075252 AF075253 AF075254 AF075255 AF075256 AF075257 AF075258 AF075259 AF093100 AF093101 AF093102 AF093103 AF093104 AF093105 AF100566 AF348335 AF348336 AF375051 AF448535 AF448536 AF448537 AF448538 AF448539 AY741139 AY823299 AY966910 AY966913 AY973944 AY986475 DQ390224 J04332 L00930 L01442 L01443 L04598 L04599 L04653 M14937 NC_001449 U34999 U55341 U55342 U55345 U55346 U55347 U55350 U55360 U55362 U82699 U96408 X04368 AF214040 AF229608 DQ393790 DQ393791 DQ393792 DQ393793 DQ393794 DQ432026 DQ432027 J03854 NC_003908 AF339479.

### SFV infectious clone

The plasmid pSP6-SFV4, transcription from which produces infectious SFV4, has been previously described [17].

### Antibodies

The following rabbit Abs (Genscript) were used: Ab-6KTF-N – raised against peptide sequence SVAETMAYLWDQNQC (SFV 6K and TF amino acids 2–15 + 'C'), and Ab-TF-C – raised against peptide sequence TEPRGNRQSLRTFDC (SFV TF amino acids 52–65 + 'C'). A rabbit anti-SFV polyclonal Ab, described in ref. [35], was also used for immunofluorescence.

### Mass spectrometry

BHK-21 cells (ATCC) were maintained and transfected by electroporation with *in vitro *transcribed RNA as described previously [17]. At 24 hr post-electroporation, SFV4 virions were concentrated from the medium by ultracentrifugation. Briefly, the medium was filtered through a 0.22 *μ*m filter before being subjected to centrifugation through a 20% sucrose cushion (20% sucrose in TNE; Tris-HCL pH 7.4, 100 mM NaCl, 0.05 mM EDTA) in a Beckman SW28 rotor (25 krpm, 2 hr, 4°C). The virus pellet was then resuspended in SDS-sample buffer and the SFV4 virion proteins were separated by SDS-PAGE (16% Tricine gels; InVitrogen), and stained with Coomassie Blue to verify purity of the sample. Gel slices containing low molecular mass products were subjected to in-gel trypsin digestion. A portion of the trypsin digest was subsequently also subjected to a mild (~20 mins) chymotrypsin digest.

LC/MS/MS data were acquired using a LTQ-FT hybrid mass spectrometer (ThermoElectron Corp). Peptide molecular masses were measured by Fourier transform-ion cyclotron resonance (FT-ICR). Peptide sequencing was performed by collision-induced dissociation (CID) in the linear ion trap of the LTQ-FT instrument. Digest samples were introduced by nanoLC (Eksigent, Inc.) with nano-electrospray ionization (ThermoElectron Corp). NanoLC was performed using a homemade C18 nanobore column (75 *μ*m i.d. × 10 cm; Atlantis C18 [Waters Corp.]; 3 *μ*m particle size). Peptides were eluted from a 50-min linear gradient run from 4% acetonitrile (with 0.1% formic acid) to 70% acetonitrile (with 0.1% formic acid).

Peptides were identified using the MASCOT search engine using a custom database including the SFV4 6K, TF and E3 sequences. The possibility of palmitoyl modifications on Cys, Lys, Ser or Thr residues was also included. Decoy searches were performed using the entire Mass Spectrometry Data Base (MSDB; i.e. all taxonomies). Such searches identified a number of other proteins (mostly contaminating keratins). However, no non-alphavirus proteins were clearly identified with peptides matching SLSFLSATEPR, TFDSNAER, SLSFLV or SLSFF (i.e. with the same molecular ions and MS/MS sequence information). There were some putative hits for the peptide molecular ions but, in each case, no other peptides were also found for the same protein – thus the probability of such assignments being real is very low.

### TF knockout mutant, TF^-^

Methylated (NEB) pSP6-SFV4 was used as template for PCR using KOD polymerase (Novagen) and primers 41TF (GCCTTTCTTTTTTAGTGCTACTTAGCCTCGGGGC) and 41TR (GCCCCGAGGCTAAGTAGCACTAAAAAAGAAAGGC). PCR product was then transformed into DH5αT1 cells (InVitrogen) and plated on Ampicillin-LB plates. Propagated plasmids were sequenced with primers 6KF (ATATCGATCTTCGCGTCG) and 6KR (ACCGTCTTGTACTCACAG) prior to *in vitro *transcription.

### Plaque assays

BHK-21 cells were transfected by electroporation with SFV4 or TF^- ^RNA in triplicate and incubated for 24 hr. Virus release into the supernatant was quantified by plaque assay. BHK cells were infected with 10-fold dilutions of each supernatant for 1 hr. The virus innoculum was then removed and cells were overlayed with 1.8% agarose containing medium. After 48 hr incubation, cells were fixed with 4% formalin and plaques were visualized with crystal violet.

### Pulse chase

Metabollic labelling of BHK-21 cells transfected with SFV4 or TF^- ^was done essentially as described previously [8]. Cells were pulsed with [^35^S]Met/Cys (Perkin Elmer) for 30 min, 8 hr post-electroporation. Cells were then incubated in growth medium containing 10-fold excess of unlabelled methionine and cysteine for 1 or 15 hr. The supernatents were then collected and the cells were lysed with a Triton X-100 containing buffer. The presence of 6K and TF in lysates was determined by immunoprecipitation with Ab-6KTF-N or Ab-TF-C, followed by SDS-PAGE (10–20% Tricine gradient gels; InVitrogen).

Radiolabelled virions in the supernatants were concentrated by ultracentrifugation as described above (Beckman SW40 rotor, 30 krpm, 2 hr, 4°C). Concentrated virions were then either directly solubilized in SDS-sample buffer and analyzed by SDS-PAGE or the Triton X-100 containing lysis buffer and analyzed by immunoprecipation as above. Radiolabelled 6K and TF proteins were quantified using a Storm Phospho-Imager and ImageJ 1.40f software.

### Immunofluorescence

Cells transfected with SFV4 or TF^- ^were grown on glass coverslips and fixed with acetone or 4% paraformaldehyde 15 hr post-electroporation. Cells were blocked in 5% mouse serum prior to incubation with rabbit polyclonal anti-SFV Ab, Ab-6KTF-N or Ab-TF-C overnight at 4°C. After washing, cells were then incubated with biotinylated mouse anti-rabbit IgG (Sigma) for 2 hr followed by incubation with Streptavadin-FITC (DAKO). Cells on coverslips were then mounted in a DAPI containing mounting medium (Vector Labs) and analyzed by fluorescence microscopy.

## List of abbreviations

AURAV: Aura virus; BEBV: Bebaru virus; BFV: Barmah Forest virus; CHIKV: Chikungunya virus; EEEV: Eastern equine encephalitis virus; FMV: Fort Morgan virus; GETV: Getah virus; HJV: Highlands J virus; MAYV: Mayaro virus; MIDV: Middelburg virus; NDUV: Ndumu virus; NSAV: Norwegian salmonid alphavirus; ONNV: O'nyong-nyong virus; RRV: Ross River virus; SAGV: Sagiyama virus; SDV: Sleeping disease virus; SESV: Seal louse virus; SFV: Semliki forest virus; SINV: Sindbis virus; SPDV: Salmon pancreas disease virus; TROV: Trocara virus; UNAV: Una virus; VEEV: Venezuelan equine encephalitis virus; WEEV: Western equine encephalomyelitis virus; WHAV: Whataroa virus.

## Competing interests

The authors declare that they have no competing interests.

## Authors' contributions

AEF carried out the bioinformatic analyses and wrote the manuscript. BYWC and MNF carried out the experimental analyses and wrote the methods section. All authors analyzed data and edited and approved the final manuscript.

## Appendix 1

In light of the results presented in this manuscript, it is important to revisit and reinterpret many earlier results regarding the 6K doublet. The fact that '6K' is present in two forms was first demonstrated and investigated by Gaedigk-Nitschko & Schlesinger [7]. The authors concluded that one form, which they labelled '4K', was a partially acylated form of the other, which they labelled '6K'. We have proposed instead that '4K' equates to 6K and '6K' is in fact TF, both of which may be acylated to varying degrees. In the following, '6K' and '4K' refer to the labels in ref. [7], while TF and 6K refer to the proteins as defined in this paper. Note that, in SINV, 6K (6.2 kDa) has five Cys residues while TF (8.0 kDa) has nine Cys residues, thus providing TF with even more potential palmitoylation sites than 6K. In SFV, both 6K (6.6 kDa) and TF (8.3 kDa) have five Cys residues.

In studies with SINV, ref. [7] showed that '4K' and '6K' have the same N-term (using Abs to a 16 amino acid N-term peptide and, in addition, by using radiolabelling of Phe at amino acid 3 and Met at amino acid 7). They also showed that both '4K' and '6K' have a Lys residue near the C-term and in fact, in SINV, both 6K and TF do (Figure 5). Additionally, with reference to excluded data, they showed that both '4K' and '6K' lack any His residues (in order to demonstrate that '6K' was not an extension into E1, which contains an N-term proximal His). This, however, is in disagreement with our findings, since TF contains a His residue (Figure 5).

At such low molecular masses, migration can depend strongly on the exact amino acid sequence and/or post-translational modifications. Thus the masses '4K' and '6K' may be unreliable. Indeed, in Figure 4A and 4C of ref. [7], the molecular mass of '6K' appears closer to 10–12 kDa on the marker scale, which may be more in keeping with an (acylated) TF than 6K. In Figure 4C (lane 2) of ref. [7], if the only difference between '6K' and '4K' is the degree of acylation, then the deacylated '6K' and '4K' should migrate together (as lane 1 of Figure 4B appears to show that deacylation is complete), which they do not. If, however, the comparison is between acylated 6K and TF and deacylated 6K and TF, then the migration patterns seen in this figure make sense. A similar interpretation explains the migration patterns seen in Figure 2 of ref. [16], in which WT SINV (lane 1) is compared to a mutant virus (lane 5) in which four of the Cys residues in 6K have been replaced with other amino acids.

Figure 4A of ref. [7], as well as Figure 2A and 2B of ref. [15], show that only '6K' (i.e. TF) is in the virion. Table 1 of ref. [7] shows that ~2.5× as many '4K' as '6K' are present in SINV-infected cells. This calculation is based on [^35^S]Cys labelling and assumes that both '4K' and '6K' contain 5 Cys residues. If in fact '6K' is TF, then it contains 9 Cys residues, so the inferred '6K' abundance must be multiplied by 5/9. Hence we propose that there are in fact 4.5× as many '4K' as '6K' in SINV-infected cells, which implies a frameshift efficiency of ~18% (in ref. [16], however, the '6K':'4K' ratio is given as 0.2, which translates to a frameshift efficiency of ~10%). These figures are similar to our own findings (10–17% with dual luciferase assay for WT SINV and SFV inserts, and ~15% for [^35^S]Met/Cys-labelled SFV-infected cell lysate), and other literature, where the more slowly migrating band of the 6K doublet is always much fainter than the more quickly migrating band [7,15,16,21]. In particular, in Figure 2 of ref. [16], for a mutant virus (lane 5) in which four of the Cys residues in 6K have been replaced with other amino acids, the bands for TF (5 remaining Cys) and 6K (1 remaining Cys) assume similar intensities when labelled with [^35^S]Cys, as expected for a frameshift efficiency of ~18%.

Figure 4A and Figure 3A of ref. [7] show that both '4K' and '6K' are acylated. The relative intensities of the 6K and TF bands when labelled with [^3^H]palmitic acid and when labelled with [^35^S]Cys (lanes 1 and 2 of Figure 4A) indicate that TF is much more heavily palmitoylated than 6K. Indeed the authors estimated that SINV '6K' carries 3–4 fatty acids (which may translate to 5–7 for TF) and that '4K' (i.e. 6K) carries just one fatty acid. The difference in the relative intensities of the [^3^H]palmitic acid-labelled 6K and TF bands between SINV and SFV in Figure 3C (lanes 1 and 2) of ref. [7] is consistent with the TF:6K Cys ratio being 9:5 in SINV but just 5:5 in SFV (i.e. SINV TF may be more heavily palmitoylated than SFV TF). Similarly, the fact that the SINV TF band migrates behind the SFV TF band Figure 3C (lanes 1 and 2) of ref. [7], despite the unmodified SINV TF peptide sequence having a slightly lower molecular mass than the unmodified SFV TF peptide sequence, is consistent with SINV TF bearing more fatty acids than SFV TF. Furthermore, Figure 4C (lane 2) of ref. [7] shows that the molecular mass of TF is significantly reduced upon deacylation with hydroxylamine, but any change in the molecular mass of 6K upon deacylation appears to be much less pronounced – again consistent with 6K being much less palmitoylated than TF. Similar effects are seen in Figure 2 of ref. [16] for the mutant virus (lane 5) in which four of the Cys residues in 6K have been replaced with other amino acids.

## Supplementary Material

Additional file 1 Mass spectrometry traces. LC/MS/MS data for selected tryptic and chymotryptic peptides of low molecular mass products isolated from purified SFV virions. Data are presented for the following peptides: SLSFLSATEPR, TFDSNAER, SLSFL, SLSFLV and SLSFF. **A**. Primary FTMS mass spectra. **B**. Output from MASCOT software showing MS/MS fragmentation spectra and matched peptide sequences. Peptide assignments were based on a combination of the following criteria: (*i*) The mass error should be less than 3 ppm. (*ii*) A check of the primary FTMS data should confirm that the charge state of the molecular ion determined by the THERMO software is correct and consistent with the actual charge state of the ion that was searched by MASCOT. (*iii*) The MS/MS data (i.e. peptide sequence data) should be of good quality and show good signal-to-noise. The MASCOT-assigned ions should be prominent and generally the main ions accounted for in the MS/MS spectrum (not noise peaks). Mass errors for the ions in the MS/MS data should mostly be within 0.3 Da. Assigned ions in the MS/MS ion-trap data are frequently relatively minor peaks, but they should still have a reasonable signal-to-noise ratio, such as 5:1. (*iv*) The MASCOT score should usually be ≥ 20. However, the score for a given peptide is proportional to the number of fragment ions observed and assigned in the MS/MS data. Thus, for small peptides, such as SLSFL and SLSFF, the MASCOT score may be less than 20, provided the quality of the MS/MS data is still relatively good. (*v*) The 'expect' value should generally be ≤ 0.0001. However, once again, for small peptides expect values ≤ 0.01 may be acceptable, since the expect values also depend on peptide length. Click here for file

Additional file 2 Immunofluorescence of SFV-infected cells showing location of 6K and TF proteins. Green fluorescence indicates Abs binding to target peptides. Cell nuclei are stained blue. (A) Ab-TF-C – Ab to C-term of TF. (B) Ab-6KTF-N – Ab to common N-term of 6K and TF. (C) Anti-SFV Ab. Cells fixed in acetone are permeabilized, allowing intracellular Ab staining. Cells fixed in 4% PFA are not permeabilized, thus only allowing Abs to bind to peptides at the cell surface. Cells are infected with WT SFV4 virus (WT), the TF knockout mutant (TF^-^), or are non-infected controls. TF^- ^also serves as an additional control for Ab-TF-C. Click here for file
