# Supplementary material for: Discovery of frameshifting in Alphavirus 6K resolves a 20-year enigma
Source: Virol J. 2008 Sep 26;5:108. doi: 10.1186/1743-422X-5-108 (PMC2569925; doi:10.1186/1743-422X-5-108)

Trypsin digest, peptide 'SLSFLSATEPR'.

A.

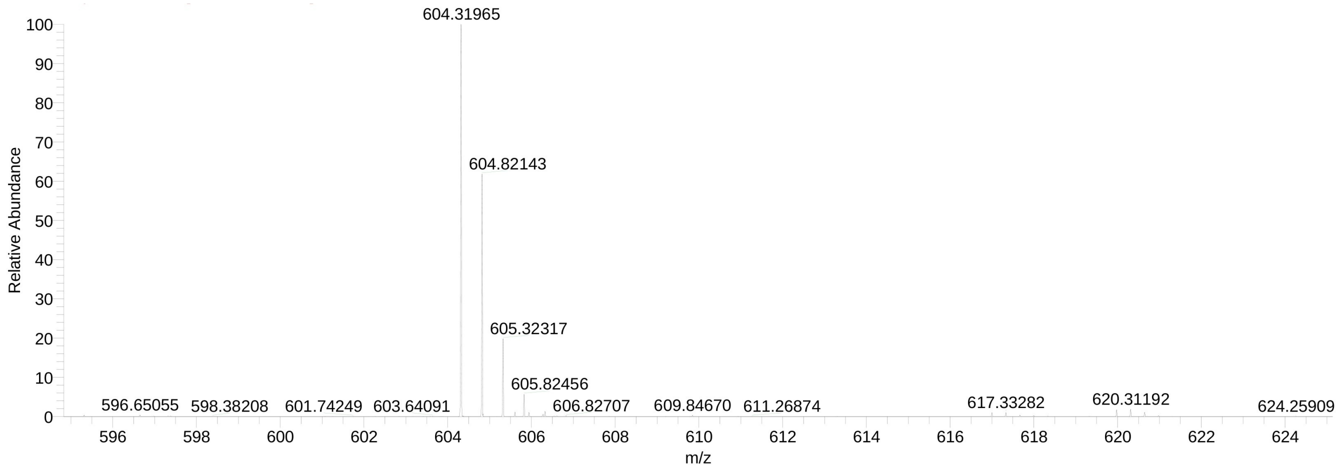

B.

MS/MS Fragmentation of **SLSFLSATEPR**

Match to Query 3516: 1206.625034 from(604.319793,2+)

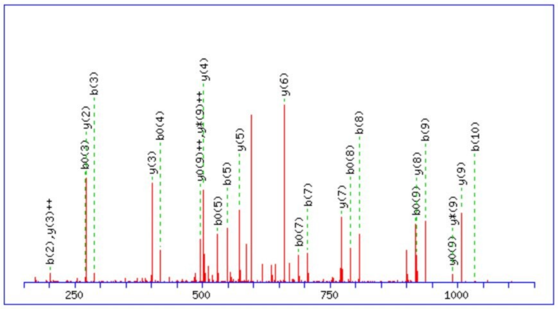

Score	Mr(calc):	Delta	Sequence
60.7	1206.6244	0.0006	SLSFLSATEPR

Monoisotopic mass of neutral peptide Mr(calc): 1206.6244
Ions Score: 61 **Expect:** 4.3e-008
Matches (Bold Red): 26/96 fragment ions using 45 most intense peaks

#	b	b^{++}	b^{0}	b^{0++}	Seq.	y	y^{++}	y*	y^{*++}	y^{0}	y^{0++}	#
1	88.0393	44.5233	70.0287	35.5180	S							11
2	**201.1234**	101.0653	183.1128	92.0600	L	1120.5997	560.8035	1103.5731	552.2902	1102.5891	551.7982	10
3	**288.1554**	144.5813	**270.1448**	135.5761	S	**1007.5156**	504.2614	**990.4891**	**495.7482**	**989.5051**	**495.2562**	9
4	435.2238	218.1155	**417.2132**	209.1103	F	**920.4836**	460.7454	903.4570	452.2322	902.4730	451.7402	8
5	**548.3079**	274.6576	**530.2973**	265.6523	L	**773.4152**	387.2112	756.3886	378.6980	755.4046	378.2059	7
6	635.3399	318.1736	617.3293	309.1683	S	**660.3311**	330.6692	643.3046	322.1559	642.3206	321.6639	6
7	**706.3770**	353.6921	**688.3664**	344.6869	A	**573.2991**	287.1532	556.2725	278.6399	555.2885	278.1479	5
8	**807.4247**	404.2160	**789.4141**	395.2107	T	**502.2620**	251.6346	485.2354	243.1214	484.2514	242.6293	4
9	**936.4673**	468.7373	**918.4567**	459.7320	E	**401.2143**	**201.1108**	384.1878	192.5975	383.2037	192.1055	3
10	**1033.5200**	517.2637	1015.5095	508.2584	P	**272.1717**	136.5895	255.1452	128.0762			2
11					R	175.1190	88.0631	158.0924	79.5498			1

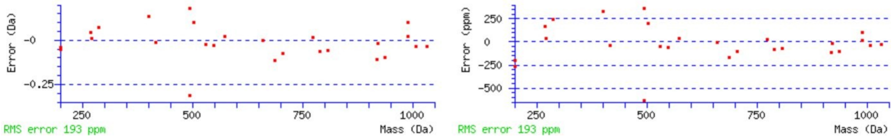

Trypsin digest, peptide 'TFDSNAER'.

A.

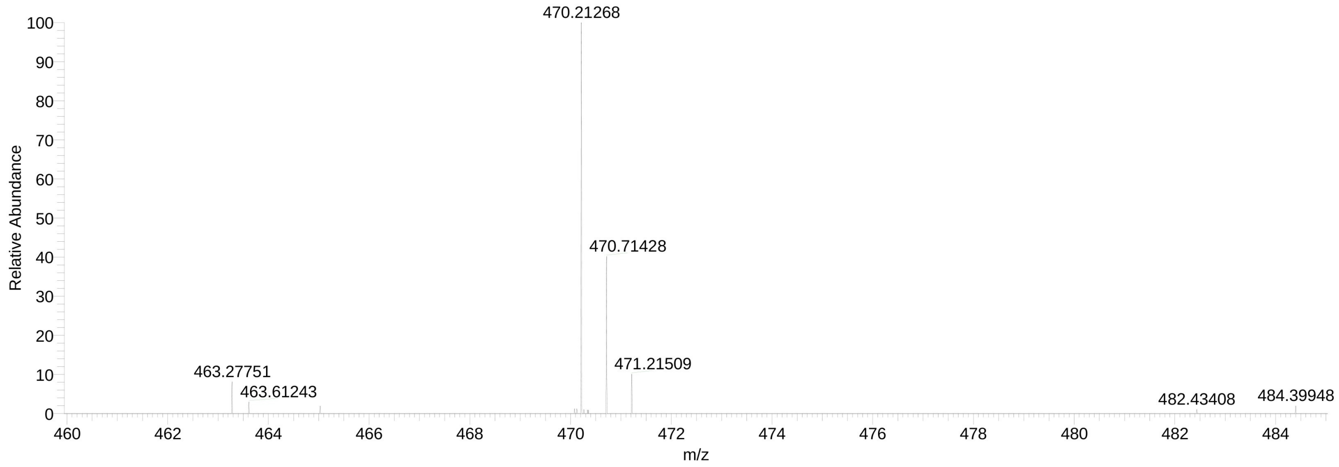

B.

MS/MS Fragmentation of **TFDSNAER**
Match to Query 2625: 938.411354 from(470.212953,2+)

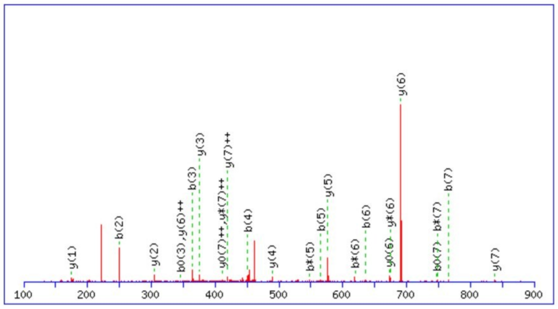

Score	Mr(calc):	Delta	Sequence
47.4	938.4093	0.0020	TFDSNAER

Monoisotopic mass of neutral peptide Mr(calc): 938.4093
Ions Score: 47 **Expect:** 3.7e-006
Matches (Bold Red): 24/74 fragment ions using 43 most intense peaks

#	b	b^{++}	b*	b^{*++}	b^0	b^{0++}	Seq.	y	y^{++}	y*	y^{*++}	y^0	y^{0++}	#
1	102.0550	51.5311			84.0444	42.5258	T							8
2	**249.1234**	125.0653			231.1128	116.0600	F	**838.3690**	**419.6881**	821.3424	**411.1748**	820.3584	**410.6828**	7
3	**364.1503**	182.5788			**346.1397**	173.5735	D	**691.3006**	**346.1539**	**674.2740**	337.6406	**673.2900**	337.1486	6
4	**451.1823**	226.0948			433.1718	217.0895	S	**576.2736**	288.6404	559.2471	280.1272	558.2630	279.6352	5
5	**565.2253**	283.1163	**548.1987**	274.6030	547.2147	274.1110	N	**489.2416**	245.1244	472.2150	236.6112	471.2310	236.1191	4
6	**636.2624**	318.6348	**619.2358**	310.1216	618.2518	309.6295	A	**375.1987**	188.1030	358.1721	179.5897	357.1881	179.0977	3
7	**765.3050**	383.1561	**748.2784**	374.6428	**747.2944**	374.1508	E	**304.1615**	152.5844	287.1350	144.0711	286.1510	143.5791	2
8							R	**175.1190**	88.0631	158.0924	79.5498			1

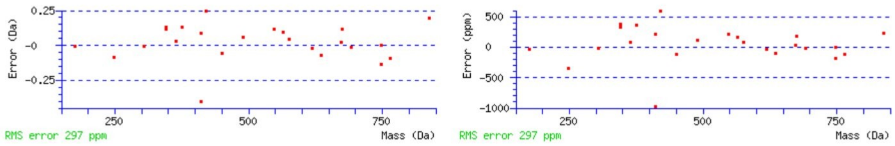

Chymotrypsin digest, peptide 'SLSFL'.

A.

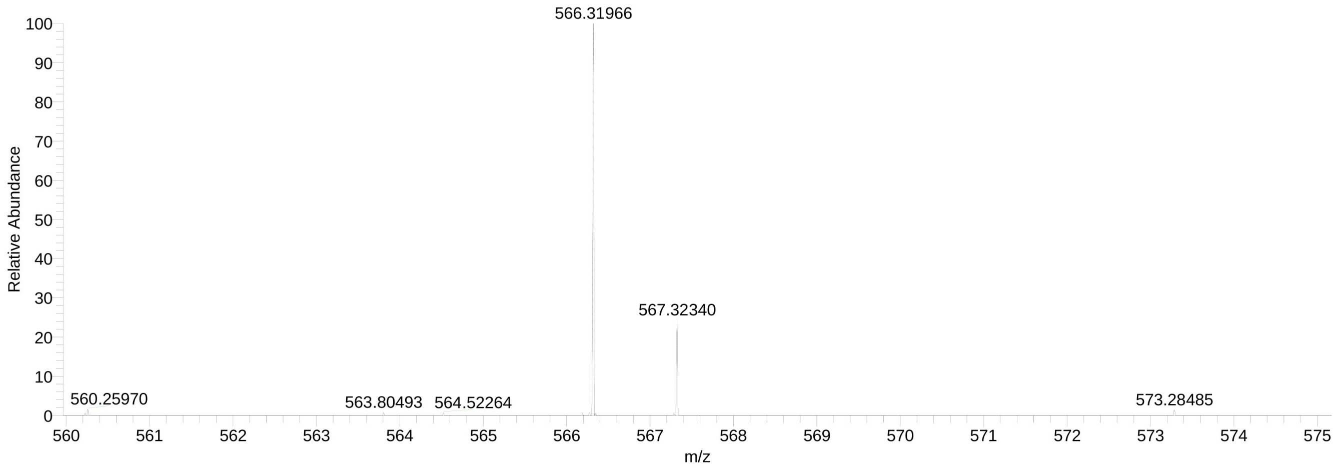

B.

MS/MS Fragmentation of **SLSFL**

Match to Query 581: 565.312484 from(566.319760,1+)

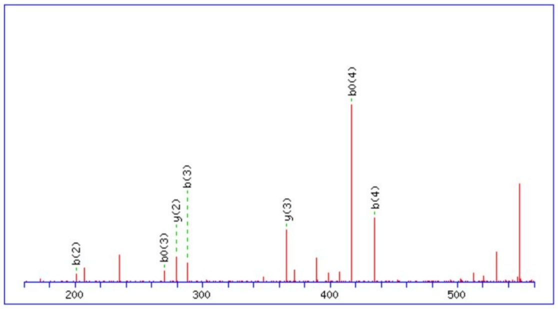

Monoisotopic mass of neutral peptide Mr(calc): 565.3111
Ions Score: 24 **Expect:** 0.00065
Matches (Bold Red): 7/14 fragment ions using 12 most intense peaks

#	b	b^0	Seq.	y	y^0	#
1	88.0393	70.0287	S			5
2	**201.1234**	183.1128	L	479.2864	461.2758	4
3	**288.1554**	**270.1448**	S	**366.2023**	348.1918	3
4	**435.2238**	**417.2132**	F	**279.1703**		2
5			L	132.1019		1

Score	Mr(calc):	Delta	Sequence
23.6	565.3111	0.0013	SLSFL
0.7	565.3111	0.0013	LSFLS

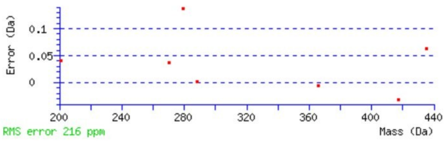

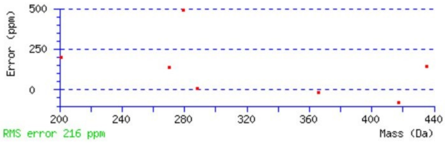

Chymotrypsin digest, peptide 'SLSFLV'.

A.

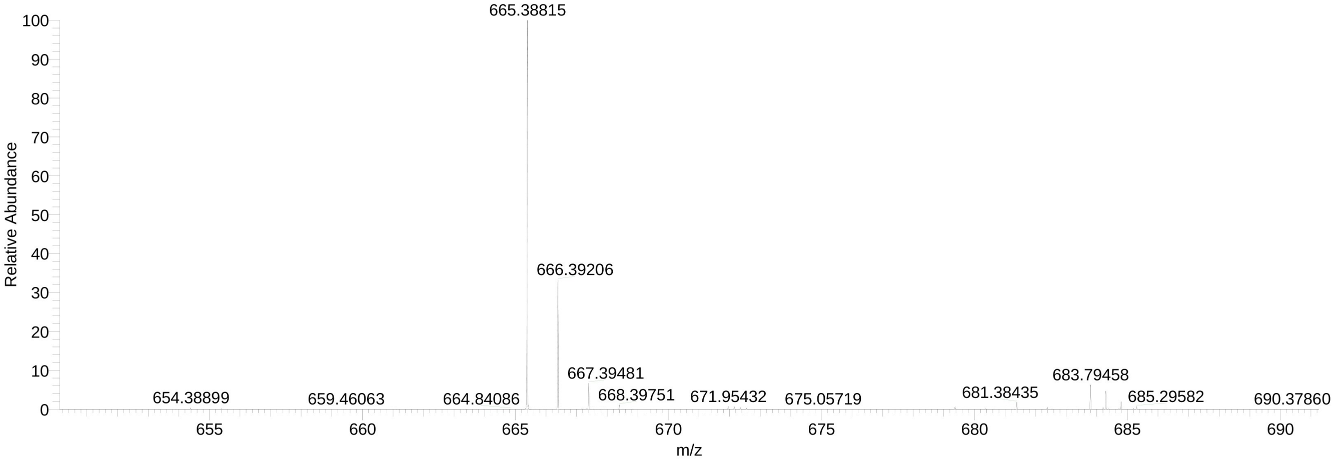

B.

MS/MS Fragmentation of **SLSFLV**

Match to Query 1461: 664.381274 from(665.388550,1+)

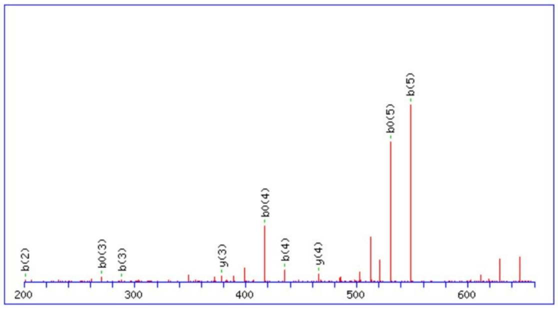

Monoisotopic mass of neutral peptide Mr(calc): 664.3795
Ions Score: 27 **Expect:** 0.00011
Matches (Bold Red): 9/17 fragment ions using 21 most intense peaks

#	b	b^0	Seq.	y	y^0	#
1	88.0393	70.0287	S			6
2	**201.1234**	183.1128	L	578.3548	560.3442	5
3	**288.1554**	**270.1448**	S	**465.2708**	447.2602	4
4	**435.2238**	**417.2132**	F	**378.2387**		3
5	**548.3079**	**530.2973**	L	231.1703		2
6			V	118.0863		1

Score	Mr(calc):	Delta	Sequence
26.6	664.3795	0.0017	SLSFLV

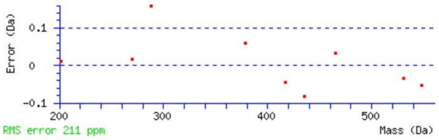

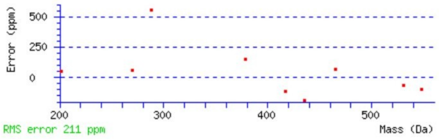

Trypsin digest, peptide 'SLSFLV'.

A.

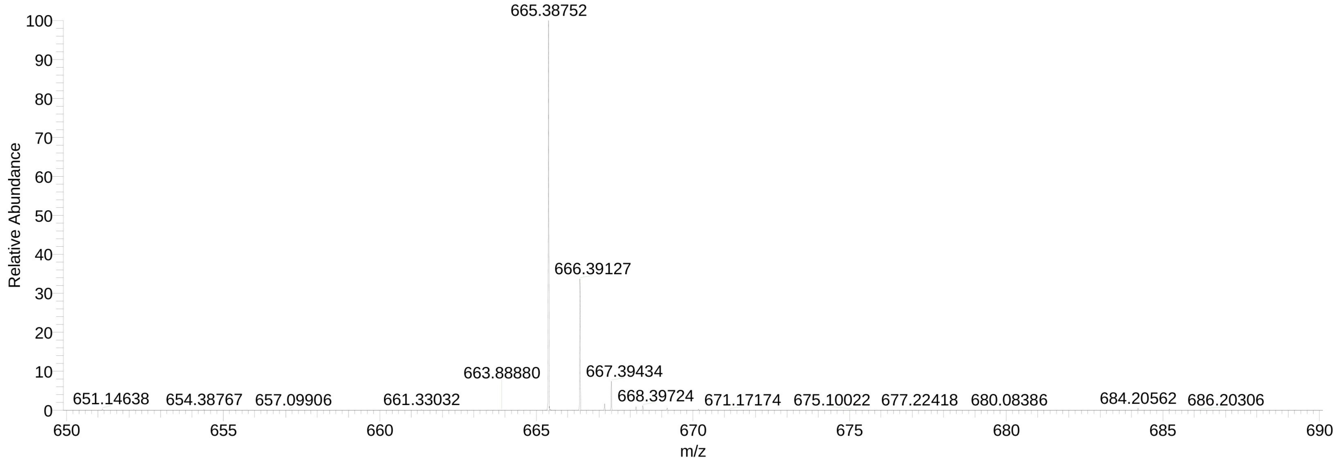

B.

MS/MS Fragmentation of **SLSFLV**
Match to Query 920: 664.380354 from(665.387630,1+)

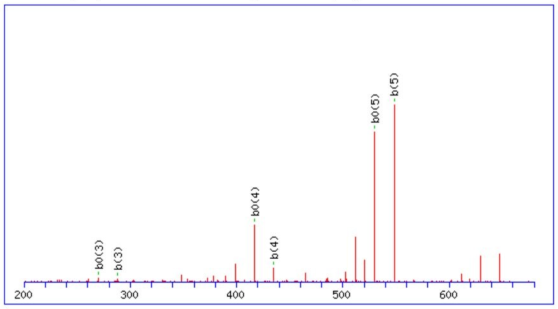

Monoisotopic mass of neutral peptide Mr(calc): 664.3795
Ions Score: 25 **Expect:** 0.00015
Matches (Bold Red): 6/17 fragment ions using 12 most intense peaks

#	b	b^0	Seq.	y	y^0	#
1	88.0393	70.0287	S			6
2	201.1234	183.1128	L	578.3548	560.3442	5
3	**288.1554**	**270.1448**	S	465.2708	447.2602	4
4	**435.2238**	**417.2132**	F	378.2387		3
5	**548.3079**	**530.2973**	L	231.1703		2
6			V	118.0863		1

Score	Mr(calc):	Delta	Sequence
25.1	664.3795	0.0008	SLSFLV

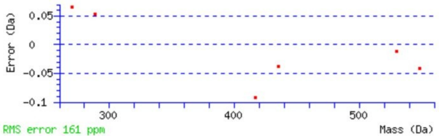

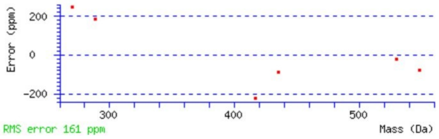

Trypsin digest, peptide 'SLSFF'.

A.

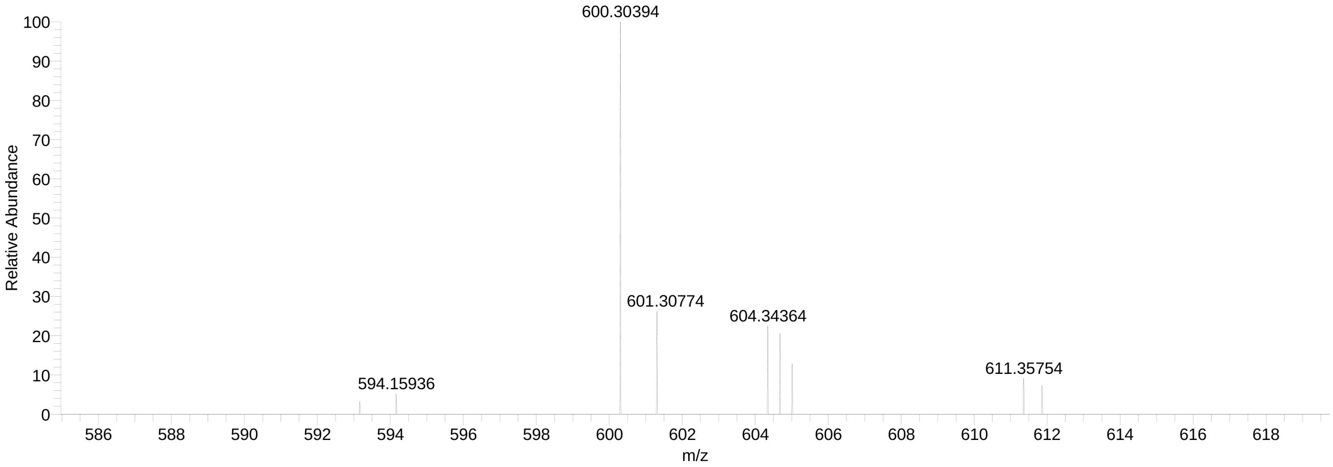

B.

MS/MS Fragmentation of **SLSFF**

Match to Query 545: 599.295944 from(600.303220,1+)

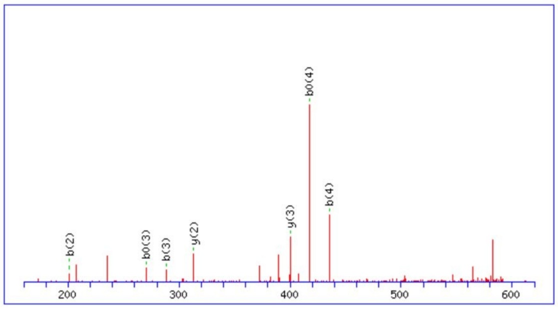

Monoisotopic mass of neutral peptide Mr(calc): 599.2955
Ions Score: 15 **Expect:** 0.0034
Matches (Bold Red): 7/14 fragment ions using 20 most intense peaks

#	b	b^0	Seq.	y	y^0	#
1	88.0393	70.0287	S			5
2	**201.1234**	183.1128	L	513.2708	495.2602	4
3	**288.1554**	**270.1448**	S	**400.1867**	382.1761	3
4	**435.2238**	**417.2132**	F	**313.1547**		2
5			F	166.0863		1

Score	Mr(calc):	Delta	Sequence
14.7	599.2955	0.0005	SLSFF

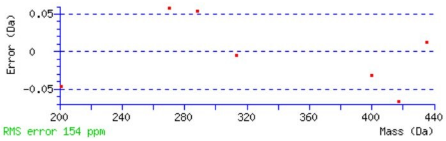

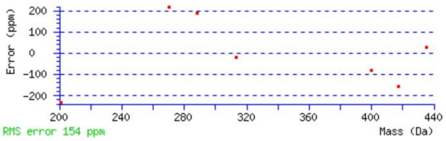

Supplement: Additional file 1 — Mass spectrometry traces. LC/MS/MS data for selected tryptic and chymotryptic peptides of low molecular mass products isolated from purified SFV virions. Data are presented for the following peptides: SLSFLSATEPR, TFDSNAER, SLSFL, SLSFLV and SLSFF. A. Primary FTMS mass spectra. B. Output from MASCOT software showing MS/MS fragmentation spectra and matched peptide sequences. Peptide assignments were based on a combination of the following criteria: (i) The mass error should be less than 3 ppm. (ii) A check of the primary FTMS data should confirm that the charge state of the molecular ion determined by the THERMO software is correct and consistent with the actual charge state of the ion that was searched by MASCOT. (iii) The MS/MS data (i.e. peptide sequence data) should be of good quality and show good signal-to-noise. The MASCOT-assigned ions should be prominent and generally the main ions accounted for in the MS/MS spectrum (not noise peaks). Mass errors for the ions in the MS/MS data should mostly be within 0.3 Da. Assigned ions in the MS/MS ion-trap data are frequently relatively minor peaks, but they should still have a reasonable signal-to-noise ratio, such as 5:1. (iv) The MASCOT score should usually be ≥ 20. However, the score for a given peptide is proportional to the number of fragment ions observed and assigned in the MS/MS data. Thus, for small peptides, such as SLSFL and SLSFF, the MASCOT score may be less than 20, provided the quality of the MS/MS data is still relatively good. (v) The 'expect' value should generally be ≤ 0.0001. However, once again, for small peptides expect values ≤ 0.01 may be acceptable, since the expect values also depend on peptide length. [file 1743-422X-5-108-S1.pdf]
