# Supplementary figures and images for: Discovery of frameshifting in Alphavirus 6K resolves a 20-year enigma
Source: Virol J. 2008 Sep 26;5:108. doi: 10.1186/1743-422X-5-108 (PMC2569925; doi:10.1186/1743-422X-5-108)

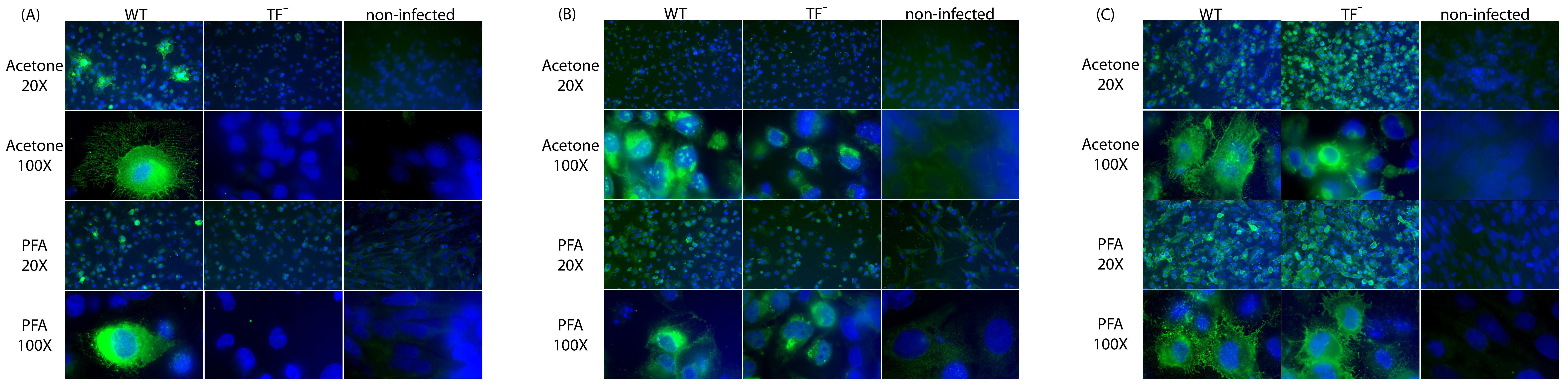

Supplement: Additional file 2 — Immunofluorescence of SFV-infected cells showing location of 6K and TF proteins. Green fluorescence indicates Abs binding to target peptides. Cell nuclei are stained blue. (A) Ab-TF-C – Ab to C-term of TF. (B) Ab-6KTF-N – Ab to common N-term of 6K and TF. (C) Anti-SFV Ab. Cells fixed in acetone are permeabilized, allowing intracellular Ab staining. Cells fixed in 4% PFA are not permeabilized, thus only allowing Abs to bind to peptides at the cell surface. Cells are infected with WT SFV4 virus (WT), the TF knockout mutant (TF-), or are non-infected controls. TF- also serves as an additional control for Ab-TF-C. [file 1743-422X-5-108-S2.jpeg]
